# Probing the impact of sulfur/selenium/carbon linkages on prodrug nanoassemblies for cancer therapy

**DOI:** 10.1038/s41467-019-11193-x

**Published:** 2019-07-19

**Authors:** Bingjun Sun, Cong Luo, Xuanbo Zhang, Mengran Guo, Mengchi Sun, Han Yu, Qin Chen, Wenqian Yang, Menglin Wang, Shiyi Zuo, Pengyu Chen, Qiming Kan, Haotian Zhang, Yongjun Wang, Zhonggui He, Jin Sun

**Affiliations:** 1 Department of Pharmaceutics, Wuya College of Innovation, Shenyang Pharmaceutical University, Shenyang, 110016 China; 2 Department of Pharmacy, Cancer Hospital of China Medical University, Liaoning Cancer Hospital & Institute, Shenyang, 110042 China; 3 Key Laboratory of Structure-Based Drug Design and Discovery, Ministry of Education, Shenyang Pharmaceutical University, Shenyang, 110016 China; 4 School of Life Science and Biopharmaceutics, Shenyang Pharmaceutical University, Shenyang, 110016 China

**Keywords:** Cancer microenvironment, Drug delivery, Nanomedicine

## Abstract

Tumor cells are characterized as redox-heterogeneous intracellular microenvironment due to the simultaneous overproduction of reactive oxygen species and glutathione. Rational design of redox-responsive drug delivery systems is a promising prospect for efficient cancer therapy. Herein, six paclitaxel-citronellol conjugates are synthesized using either thioether bond, disulfide bond, selenoether bond, diselenide bond, carbon bond or carbon-carbon bond as linkages. These prodrugs can self-assemble into uniform nanoparticles with ultrahigh drug-loading capacity. Interestingly, sulfur/selenium/carbon bonds significantly affect the efficiency of prodrug nanoassemblies. The bond angles/dihedral angles impact the self-assembly, stability and pharmacokinetics. The redox-responsivity of sulfur/selenium/carbon bonds has remarkable influence on drug release and cytotoxicity. Moreover, selenoether/diselenide bond possess unique ability to produce reactive oxygen species, which further improve the cytotoxicity of these prodrugs. Our findings give deep insight into the impact of chemical linkages on prodrug nanoassemblies and provide strategies to the rational design of redox-responsive drug delivery systems for cancer therapy.

## Abstract (web summary)

Prodrug-based self-assembled nanoparticles have emerged as an efficient drug delivery system (DDS) for cancer therapy. Here, the authors show that the type of bond in prodrug assemblies influences the efficiency of the DDS on several different levels.

## Introduction

Chemotherapy is the most common treatment for cancer^1^. However, the delivery efficiency of chemotherapeutic agents to tumors is far from satisfactory, resulting in limited clinical efficacy and serious adverse reactions^2^. A wide range of drug delivery strategies have been developed to address these problems. Among them, prodrug-based self-assembled nanoparticles (NPs), which integrate the advantages of prodrug strategies and nanocarriers, have emerged as an efficient drug delivery system (DDS) for cancer therapy^3,4^. In prodrug nanoassemblies, prodrug strategies could effectively improve the undesirable physicochemical properties of chemotherapeutic agents, such as poor stability, low solubility and nonselective toxicity^5^. Additionally, self-assembly to form NPs also offers distinct advantages, including extended systemic circulation, improved tumor targeting, facilitated cellular uptake, and controlled drug release^6^. More importantly, compared with conventional nano-DDSs (e.g., liposomes, micelles and nanoemulsions), prodrug nanoassemblies exhibit unique superiority in terms of high drug-loading capacity and low carrier materials-related toxicity because the prodrugs themselves serve as both the carriers and the cargos^3,4^.

In addition, specific tumor stimuli-responsive DDSs, which respond to the characteristic biological stimuli that distinguish tumors from normal tissues, have been widely investigated to facilitate the precise delivery of chemotherapeutic agents to tumors^7,8^. Compared with normal cells, most tumor cells exhibit a redox-heterogeneous intracellular microenvironment due to the simultaneous overproduction of reactive oxygen species (ROS) and glutathione (GSH)^9,10^. This redox heterogeneity may exist in different tumors or in different regions and different growth stages in one tumor^11,12^. This redox potential difference has been well studied to achieve on-demand drug release in tumor cells^13,14^. Sulfur (S)-based chemical linkages have been widely utilized to design tumor-specific, redox-responsive DDSs, including thioether bond, disulfide bond and thioketal bond^11,12,15,16^. Inspired by the redox-responsivity of sulfur bonds, selenium (Se), belonging to the same family as S in the periodic table of the elements, has also attracted increasing interest. Selenium is a unique, essential trace element that plays an important role in the antioxidant defense and redox balance of cells^17^. Recently, a series of selenoether- or diselenide-containing polymers have been developed to produce a redox-responsive DDSs for cancer therapy^18,19^.

Despite the rapid development of polymer materials, the application of selenium bonds in the fields of prodrugs and prodrug nanoassemblies has not yet been reported. There are certain differences between sulfur bonds and selenium bonds in terms of bond angles/dihedral angles, redox-responsivity and antitumor activity: (i) it was found that the bond angles/dihedral angles of disulfide bonds could significantly improve space flexibility, thus facilitating the self-assembly and enhancing the stability of nanoassemblies or proteins^20–26^. Therefore, the bond angles/dihedral angles of sulfur/selenium-based linkages might influence the self-assembly performance and colloidal stability of prodrug nanoassemblies; (ii) the difference in redox-responsivity between sulfur/selenium bonds could have remarkable influence on the drug release characteristics and cytotoxicity of prodrug nanoassemblies^27–29^; (iii) several selenium-containing molecules have been reported with the unique ability to produce ROS and induce apoptosis of tumor cells^17,30–32^, which might further affect the cytotoxicity and antitumor efficiency of prodrug nanoassemblies. Based on these considerations, we proposed that the distinct properties of sulfur/selenium bonds might significantly affect the drug delivery characteristics of prodrug nanoassemblies, including self-assembly performance, redox-responsive drug release and antitumor efficiency.

**Fig. 1:**
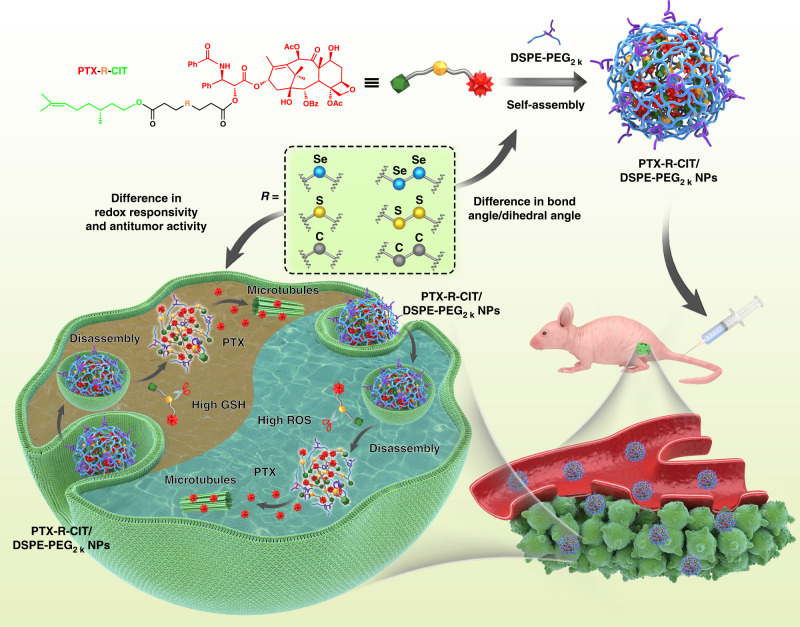
Schematic representation. Sulfur/selenium/carbon bond-bridged PTX–CIT prodrug nanoassemblies for cancer therapy

Here we show four redox-responsive prodrugs by conjugating paclitaxel (PTX) with citronellol (CIT) using thioether bond, disulfide bond, selenoether bond and diselenide bond-containing carbon chains as linkages, abbreviated as PTX–S-CIT, PTX–SS-CIT, PTX–Se-CIT and PTX-SeSe-CIT, respectively (Fig. 1). Conjugates containing same number of non-responsive carbon chains are synthesized as negative controls (PTX–C–CIT and PTX–CC–CIT). As shown in Fig. 1, the prodrugs could self-assemble into uniform sized NPs, with high drug-loading capacity (over 50%, wt%). The differences among sulfur/selenium/carbon bonds in terms of bond angle/dihedral angle, redox-responsivity and antitumor activity are compared systematically. Furthermore, the influence of sulfur/selenium/carbon bonds on the self-assembly, colloidal stability, redox-responsive drug release, cytotoxicity, pharmacokinetics, biodistribution, and antitumor efficiency of prodrug nanoassemblies are also investigated.

## Results and discussion

### Synthesis of PTX–CIT prodrugs

The routes of synthesis for sulfur/selenium/carbon bond-bridged PTX–CIT prodrugs were shown in Supplementary Figure 1, and the chemical structures of these prodrugs were confirmed by mass spectrum (MS) and ^1^H NMR (Supplementary Figures 2–7).

### Preparation and characterization of prodrug nanoassemblies

As shown in Supplementary Table 1, the hydrophobic PTX–CIT conjugates spontaneously assembled into uniform NPs in aqueous solution using the one-step nanoprecipitation method. However, the non-PEGylated prodrug nanoassemblies, with highly hydrophobic surfaces, had poor stability in PBS with significantly increased particle sizes (Supplementary Table 1). In our previous study, non-PEGylated prodrug nanoassemblies were found to be easily phagocytosed by the reticuloendothelial system (RES), leading to poor pharmacokinetic behavior and antitumor efficiency^11^. Therefore, DSPE-PEG_2K_ was used for PEGylation to achieve longer systemic circulation times of the NPs in vivo. The prodrug nanoassemblies showed spherical structures with diameters of ~90 nm (Supplementary Figure 8 and Supplementary Table 2). As the prodrugs themselves act as both the carriers and the cargos, the self-assembled NPs had ultrahigh drug-loading capacity (over 50%, wt%) and exhibited distinct superiority over conventional PTX-encapsulated nanoformulations (usually less than 10%, wt%, Supplementary Table 2).

### Colloidal stability and molecular dynamics simulations

As shown in Supplementary Table 1, the particle sizes of non-PEGylated prodrug nanoassemblies significantly increased after dilution with an equal volume of pH 7.4 PBS containing 10% FBS. The stability of non-PEGylated prodrug nanoassemblies followed the order of PTX-SeSe-CIT NPs (233.9 nm) > PTX–SS-CIT NPs (776.0 nm) > PTX–Se–CIT NPs (803.0 nm) > PTX–S–CIT NPs (1191.0 nm) > PTX–CC–CIT NPs/PTX–C–CIT NPs (>1500 nm). By contrast, PEGylated prodrug nanoassemblies showed much better colloidal stability than non-PEGylated prodrug nanoassemblies under the same conditions. As shown in Supplementary Figure 9, sulfur/selenium bond-bridged prodrug nanoassemblies, especially PTX–SeSe–CIT NPs, exhibited good colloidal stability, with negligible changes in particle size after a 24 h incubation period. In comparison, PTX–CC–CIT NPs and PTX–C–CIT NPs quickly increased in particle size during incubation. The only differences among these conjugates lie in the sulfur/selenium/carbon linkages. Therefore, these chemical linkages impose significant influence on the assembly performance and colloidal stability of prodrug nanoassemblies. It has been reported that disulfide bonds between cysteine residues play an important role in the stability of proteins^23,26^. The bond angle/dihedral angle of disulfide bonds may significantly improve space flexibility, thereby facilitating the self-assembly and enhancing the stability of nanoassemblies or proteins^20–26^. The most stable conformation has been found to correspond to the bond angle/dihedral angle of 90^°^^20,23–25^. Therefore, we calculated the bond angles of sulfur/selenium/carbon bonds and the dihedral angles of C–CC–C, C–SS–C and C–SeSe-C using the Material Studio software package. The bond angles of sulfur/selenium/carbon bonds in PTX–CIT prodrugs were shown in Supplementary Figures 10–15: –SeSe– (89.9°/93.3°); –SS– (94.6°/97.9°); -Se- (95.4°); –S– (97.8°); –CC– (111.6°/115.1°); –C– (112.6°). Notably, the bond angle of -SeSe- was closest to 90°, which may provide sufficient structural flexibility for PTX-SeSe-CIT conjugates to balance intermolecular forces and establish a favorable conformation during self-assembly^21,22^. In comparison, the bond angles of carbon linkages (–C– and –CC–) were much larger than those of sulfur/selenium bonds, resulting in limited structural flexibility unfavorable to self-assembly. Further, there were also significant differences in the dihedral angles of the diatomic linkages (-CC-, -SS- and –SeSe–). Compared with that of C–CC–C (177.4°) and C–SS–C (164.5°), the dihedral angle of C–SeSe–C was much closer to 90° (106.5°), which may further facilitate the self-assembly process and improve the colloidal stability of prodrug nanoassemblies^20^.

Molecular docking was used to investigate molecular interaction during the self-assembly process. The interaction strength between prodrug molecules was evaluated using the binding energy. From the thermodynamic point of view, a negative free energy (ΔG < 0) indicates a favorable system of interactions. Larger potential energy absolute values indicate greater stability. As shown in Supplementary Figure 16, the calculated binding energy values were PTX–SeSe–CIT (−3.5 kcal mol^−1^), PTX–SS–CIT (−2.5 kcal mol^−1^), PTX–Se–CIT (−2.3 kcal mol^−1^), PTX–S–CIT (−1.5 kcal mol^−1^), PTX–CC–CIT (−1.4 kcal mol^−1^) and PTX–C–CIT (−0.5 kcal mol^−1^). The trend in binding affinities was consistent with the stabilities of prodrug nanoassemblies, suggesting that sulfur/selenium bonds may help to establish favorable conformations during the self-assembly process and reduce the total free energies of these systems.

### In vitro redox-responsive drug release

**Fig. 2:**
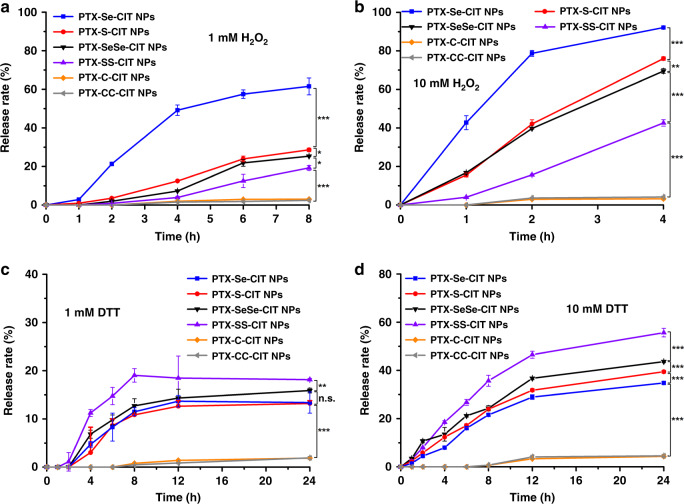
In vitro redox-responsive drug release. (**a**) 1 mM H_2_O_2_; (**b**) 10 mM H_2_O_2_; (**c**) 1 mM DTT; (**d**) 10 mM DTT. Data are presented as mean ± SD (three independent experiments). *P *<* 0.05, **P *<* 0.01, ***P *<* 0.001 by two-tailed Student’s *t* test

PTX was measured to investigate the drug release characteristics of prodrug nanoassemblies and its structure was confirmed by high resolution mass spectrum (Supplementary Figure 17). The drug release of prodrug nanoassemblies in blank media without H_2_O_2_ and DTT were shown in Supplementary Figure 18. Only a small amount of PTX (less than 10%) was released from prodrug nanoassemblies within 24 h. We then investigated the redox-responsive drug release of prodrug nanoassemblies using H_2_O_2_ (a frequently-used analogue of ROS) and dithiothreitol (DTT, a frequently-used analogue of GSH) as redox agents. In the presence of H_2_O_2_, sulfur/selenium bonds present in the prodrugs may oxidize to form hydrophilic sulfoxide/selenoxide or sulphone/selenone groups, which could facilitate the hydrolysis of the adjacent ester bond and the release of PTX from the prodrugs^16,33^. As shown in Fig. 2a, b, the sulfur/selenium bond-bridged prodrug nanoassemblies showed H_2_O_2_-trigged drug release, and the oxidation-responsivity followed the order of selenoether bond > thioether bond > diselenide bond > disulfide bond. Compared with S, Se has a larger atomic radius and weaker electronegativity, leading to a lower bond energy^34^. Therefore, selenium bond was more sensitive to H_2_O_2_ than sulfur bond^34^. Besides, the oxidation-sensitivity of -Se- and –S– were far higher than those of -SeSe- and -SS- due to the lower electronegativity. In comparison, the non-sensitive nanoassemblies (PTX–C–CIT NPs and PTX–CC–CIT NPs) exhibited extremely slow drug release even in the presence of 10 mM H_2_O_2_.

Moreover, the sulfur/selenium bond-bridged prodrug nanoassemblies also exhibited DTT-responsive drug release (Fig. 2c, d). S is directly above Se in the periodic table of the elements and has 16 electrons versus 34 for Se. Therefore, S is much more active than Se as an electron acceptor and undergoes reduction more easily^28^. As a result, nanoassemblies containing disulfide bonds were most sensitive to reduction conditions, followed by those containing diselenide bonds. In comparison, nanoassemblies containing thioether bonds and selenoether bonds showed much lower reduction-responsivity, and the carbon bond control groups were no reduction-responsive (Fig. 2c, d).

### Cytotoxicity

**Fig. 3:**
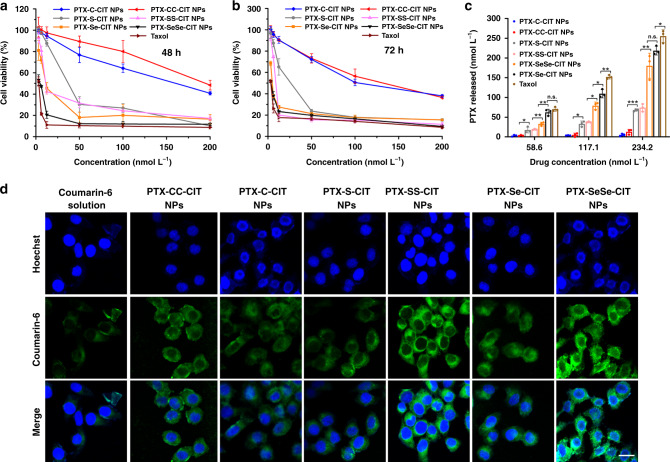
Cytotoxicity assay and cellular uptake. Viability of KB cells after treated with various concentrations of Taxol and prodrug nanoassemblies for (**a**) 48 h and (**b**) 72 h. Data are presented as mean ± SD (three independent experiments). (**c**) Free PTX released from prodrug nanoassemblies after incubation with KB cells for 48 h. Data are presented as mean ± SD (three independent experiments). *P *<* 0.05, **P *<* 0.01, ***P *<* 0.001 by two-tailed Student’s *t* test. (**d**) CLSM images of KB cells incubated with free coumarin-6 or coumarin-6-labeled prodrug nanoassemblies for 2 h. Scale bar represents 10 μm

The cytotoxicity of prodrug nanoassemblies with respect to human oral epidermoid carcinoma KB cells, human pulmonary carcinoma A549 cells and mouse breast carcinoma 4T1 cells was evaluated using the MTT assay. The half maximal inhibitory concentration (IC_50_) values were calculated and summarized in Supplementary Table 3. As shown in Supplementary Figure 19, DSPE-PEG_2K_ has good biocompatibility with KB cells. Compared with Taxol, prodrug nanoassemblies exhibited lower cytotoxicity due to the delayed release of PTX (Fig. 3a, b and Supplementary Figure 20). The cytotoxicity of prodrug nanoassemblies followed the order of PTX-SeSe-CIT NPs > PTX–Se-CIT NPs > PTX–SS-CIT NPs > PTX–S-CIT NPs » PTX–CC-CIT NPs/PTX–C-CIT NPs. It has been found that cytotoxicity is closely related to the release rate of PTX from prodrug nanoassemblies: faster drug release coincides with higher cytotoxicity. Therefore, the release of PTX from prodrug nanoassemblies was evaluated after incubation with KB cells. As shown in Fig. 3c, Taxol showed faster drug release than prodrug nanoassemblies. Much more PTX was released from PTX-SeSe-CIT NPs and PTX–Se-CIT NPs than from PTX–SS-CIT NPs and PTX–S-CIT NPs, agreeing well with the cytotoxicity results. In comparison, negligible PTX was released from PTX–CC-CIT NPs and PTX–C-CIT NPs, explaining the low proliferation inhibition observed.

The rapid release of PTX from PTX–Se-CIT NPs could be attributed to the higher oxidation-sensitivity of selenoether bond, which could quickly respond to the overproduced ROS in tumor cells. Further, as an essential regulator of the redox balance in vivo, selenium would undergo a series of redox reactions. The diselenide bond might be reduced to SeH or be oxidized to selenoxide/selenone in cells^33,35^. The selenoether bond could be oxidized to selenoxide or selenone and could then be recycled in different oxidized states^36,37^. ROS could be generated during these processes, coupled with the decline of reduced GSH and increase of oxidized GSH contents (GSSG)^31,38–40^. Therefore, we investigated the influence of prodrug nanoassemblies on the intracellular ROS, GSH and GSSG level in KB cells. As shown in Supplementary Figures 21–22, selenium-containing prodrug nanoassemblies, especially PTX-SeSe-CIT NPs, greatly increased the intracellular ROS levels of KB cells. In addition, PTX–Se-CIT NPs and PTX-SeSe-CIT NPs significantly decreased the intracellular GSH concentration and the GSH/GSSG ratio (Supplementary Figure 23). The increased intracellular oxidative stress may facilitate the oxidation-responsive release of PTX from prodrug NPs. Therefore, more PTX was released from PTX-SeSe-CIT NPs than that from PTX–SS-CIT NPs and PTX–S-CIT NPs (Fig. 3c). Besides, the selenium-induced generation of ROS and disruption of intracellular redox homeostasis could further induce apoptosis of tumor cells^39,41–43^. As a result, PTX-SeSe-CIT prodrug, with two selenium atoms and rapid drug release in tumor cells, exhibited the strongest proliferation inhibition ability.

### Cellular uptake

To investigate cellular uptake, KB cells were incubated with free coumarin-6 or coumarin-6-labeled prodrug nanoassemblies. The intracellular fluorescence intensity was measured by confocal laser scanning microscopy (CLSM) and flow cytometry. As shown in Fig. 3d and Supplementary Figures 24–26, coumarin-6-loaded prodrug nanoassemblies exhibited significantly higher intracellular fluorescence intensity than free coumarin-6 at both 0.5 and 2 h. These results indicated that prodrug nanoassemblies had higher cellular uptake efficiency.

### In vivo pharmacokinetics

**Fig. 4:**
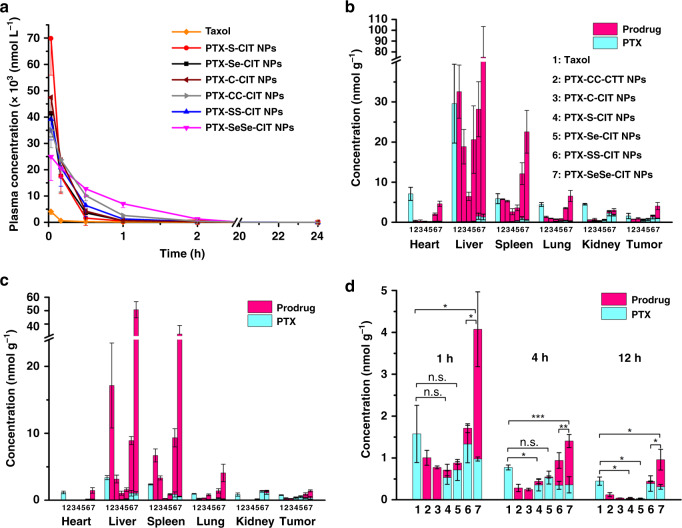
Pharmacokinetic and in vivo biodistribution. (**a**) Molar concentration-time curves of the sum of released PTX and prodrugs. Data are presented as mean ± SD (five independent experiments). (**b**, **c**) In vivo biodistribution of Taxol and prodrug nanoassemblies at 1 h (**b**) and 4 h (**c**). (**d**) Tumor accumulation of Taxol and prodrug nanoassemblies. Data are presented as mean ± SD (three independent experiments). *P *<* 0.05, **P *<* 0.01, ***P *<* 0.001 by two-tailed Student’s *t* test

Sprague-Dawley rats were used to evaluate the pharmacokinetic profiles of prodrug nanoassemblies after intravenous administration. For ease of comparison, the molar concentration-time curves were illustrated in Fig. 4a and Supplementary Figure 27, and the pharmacokinetic parameters were summarized in Supplementary Table 4. The prodrug nanoassemblies displayed significantly prolonged circulation time compared to Taxol, which was rapidly cleared from blood. Furthermore, the sulfur/selenium/carbon bonds had a great influence on the pharmacokinetic behavior. Much more PTX was released from PTX–S-CIT NPs/PTX–Se-CIT NPs than from PTX-SeSe-CIT NPs/PTX–SS-CIT NPs in oxygen-rich blood, possibly due to the superior oxidation-responsivity of thioether/selenoether bonds than disulfide/diselenide bonds. This reason also accounts for the lower area under the curve (AUC) values and shorter circulation time of PTX–Se-CIT NPs/PTX–S-CIT NPs than PTX-SeSe-CIT NPs/PTX–SS-CIT NPs. Further, PTX–CC-CIT NPs and PTX–C-CIT NPs had lower AUCs compared with PTX-SeSe-CIT NPs due to their poor colloidal stability (Supplementary Figure 9), and thus they could be quickly cleared from the body. In comparison, PTX-SeSe-CIT NPs, with the best colloidal stability and modest redox-sensitivity, exhibited the longest circulation time and highest AUC value.

### In vivo biodistribution

4T1 tumor-bearing BALB/C mice were used to evaluate the biodistribution of prodrug nanoassemblies. As shown in Fig. 4b, d and Supplementary Figure 28, prodrug nanoassemblies mainly accumulated in the liver and spleen. Notably, PTX–S-CIT NPs had a much lower concentration than other groups in normal tissues and tumors, possibly due to its high oxidation-sensitivity and poor stability. This was consistent with the pharmacokinetic behavior (Supplementary Figure 27). In comparison, PTX-SeSe-CIT NPs, with better colloidal stability and longer circulation time, showed a distinctly higher concentration than other groups in normal tissues and tumors.

### In vivo antitumor efficacy

**Fig. 5:**
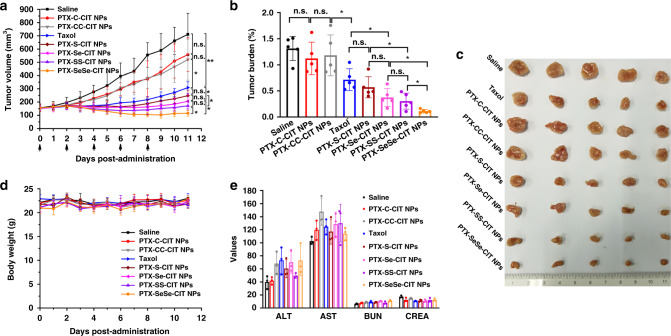
In vivo antitumor efficacy of prodrug nanoassemblies. (**a**) Tumor volume. (**b**) Tumor burden. (**c**) Images of tumors. (**d**) Body weight changes. Data are presented as mean ± SD (five independent experiments). *P *<* 0.05, **P *<* 0.01 by two-tailed Student’s *t* test. (**e**) Hepatorenal function parameters. Data are presented as mean ± SD (three independent experiments). AST: aspartate aminotransferase (U L^−1^); ALT: alanine aminotransferase (U L^−1^), BUN: blood urea nitrogen (mmol L^−1^), CREA: creatinine (μmol L^−1^)

The in vivo antitumor efficiency of prodrug nanoassemblies were evaluated in KB tumor-bearing nude mice. As shown in Fig. 5, sulfur/selenium bond-bridged prodrug nanoassemblies had more potent antitumor efficacy than Taxol and saline. In comparison, mice treated with non-sensitive PTX–CC-CIT NPs/PTX–C-CIT NPs exhibited a rapid increase in tumor volume, possibly due to the poor stability of the NPs in vivo and insufficient drug release in tumor sites. Further, PTX-SeSe-CIT NPs/PTX–SS-CIT NPs showed better tumor-inhibiting activity than PTX–S-CIT NPs/PTX–Se-CIT NPs, probably due to their better colloidal stability, longer circulation time and higher AUC values. Moreover, PTX-SeSe-CIT NPs, with multiple therapeutic advantages including good colloidal stability, long circulation time, high tumor accumulation and efficient drug release in tumor cells, exhibited the most potent tumor-inhibiting activity, with significantly reduced tumor volume. The results corroborated that in vivo antitumor efficacy is the integrated result of the above-mentioned drug delivery characteristics, and poor performance in any step of the drug delivery process would compromise the therapeutic efficacy.

The cellular apoptosis of tumor tissues was assessed using the TdT-dependent dUTP-biotin nick end labeling (TUNEL) assay. As shown in Supplementary Figure 29, sulfur/selenium bond-bridged prodrug nanoassemblies, especially PTX-SeSe-CIT NPs, caused widespread apoptosis of tumor cells, suggesting a potent antitumor efficacy. Further, sulfur/selenium bond-bridged prodrug nanoassemblies diminished the expression of Ki-67 in tumor cells, and PTX-SeSe-CIT NPs exhibited the best effect in inhibiting the tumor proliferation (Supplementary Figure 30).

No significant changes in body weight or hepatorenal function were observed in any group (Fig. 5d, e). No obvious histological damage was observed in H&E-stained tissue sections of major organs (heart, liver, spleen, lung and kidney, Supplementary Figure 31). These results suggest that the prodrug nanoassemblies were well tolerated and have negligible nonspecific toxicity to major organs and tissues.

In addition, another xenograft tumor model (4T1 tumor-bearing BALB/c mice) was used to further evaluate the antitumor efficacy of prodrug nanoassemblies. As shown in Supplementary Figures 32–35, the antitumor activity of prodrug nanoassemblies on 4T1 tumors were consistent with those observed with KB tumors, with PTX-SeSe-CIT NPs exhibiting the best tumor-inhibiting efficacy. However, the tumor volumes of the PTX–S-CIT NPs and PTX–Se-CIT NPs groups were larger than those of the Taxol group (Supplementary Figure 32a), a result that was the opposite of that observed with KB tumors. This could be attributed to the tissue distribution of prodrug nanoassemblies. As shown in Fig. 4d, PTX-SeSe-CIT NPs showed much higher tumor accumulation than the other groups, leading to a higher antitumor activity. In comparison, the molar concentrations of PTX–S-CIT NPs and PTX–Se-CIT NPs, with regards to both the prodrugs and the released PTX, were lower than the concentration of Taxol (Fig. 4d). For PTX–C-CIT NPs and PTX–CC-CIT NPs, the chemical structures of the prodrugs remained intact in tumor sites, and no PTX was released. As a result, there were no significant differences between PTX–C-CIT NPs/PTX–CC-CIT NPs and the saline group in terms of tumor volume (Supplementary Figure 32a).

In summary, six paclitaxel-citronellol prodrugs were synthesized using sulfur/selenium/carbon bonds as chemical linkages. These hydrophobic prodrugs can self-assemble into uniform size NPs with high drug loading (over 50%). The bond angle/dihedral angle, redox-responsivity and inherent antitumor activity of sulfur/selenium/carbon bonds significantly affected the drug delivery characteristics of prodrug nanoassemblies. Diselenide bond-bridged prodrug nanoassemblies exhibited the most potent antitumor activity due to its multiple therapeutic advantages, including good colloidal stability, long circulation time, high tumor accumulation and efficient drug release in tumor cells. Our findings clarified the impact of sulfur/selenium/carbon-containing linkages on the drug delivery characteristics and effectiveness of prodrug nanoassemblies, and provided a knowledge base for the rational design of redox-responsive DDS for efficient cancer therapy.

## Methods

### Materials

PTX was purchased from Jingzhu Bio-technology Co., Ltd (Nanjing, China). Taxol was purchased from Aosaikang Pharmaceutical Co., Ltd (Jiangsu, China). CIT was obtained from J&K Scientific. Ltd (Beijing, China). Selenium, sodium borohydride (NaBH_4_), DTT, H_2_O_2_, and acetic anhydride were purchased from Aladdin (Shanghai, China). 1-Ethyl-3-(3-dimethyllaminopropyl) carbodiimide hydrochloride (EDCI), hydroxybenzotriazole (HOBt) and 4-dimethylaminopyrideine (DMAP) were obtained from Pukang Pharm Co., Ltd (Zhejiang, China). Cell culture reagents were purchased from GIBCO, Invitrogen Corp. (Carlsbad, California, USA). 1,2-distearoyl-sn-glycero-3-phosphoethanolamine-N-[methoxy(polyethyleneglycol)-2000] (DSPE-PEG_2K_) was purchased from Shanghai Advanced Vehicle Technology Co., Ltd. 3-(4,5-dimethylthiazol-2-yl)-2,5-diphenyl tetrazolium bromide (MTT), 4’,6-diamidino-2-phenylindole (DAPI), trypsin-EDTA and coumarin-6 were obtained from Dalian Meilun Biotechnology Co., Ltd (Dalian, China). Hoechst 33342 was purchased from BD Biosciences, USA. DAPI was purchased from Vector laboratories, Burlingame, CA. DiR was purchased from ATT Bioquest (Beijing, China). TUNEL apoptosis detection kit was purchased from Vazyme Biotech Co., Ltd. (China). All other reagents and solvents mentioned in this article were of analytical grade.

### Synthesis of 3, 3′-Diselanediyldipropanoic acid

3,3′-Diselanediyldipropanoic acid was synthesized according to literature methods with minor modifications^27^. Selenium powder (3.16 g, 40 mmol) in 30 mL of cold water was added to a two-necked flask under a nitrogen atmosphere. NaBH_4_ (3.03 g, 80 mmol) dissolved in 20 mL of cold H_2_O was syringed dropwise into the selenium suspension. After the initial vigorous reaction had subsided (30 min), another quantity of selenium powder (3.16 g, 40 mmol) was added, and the mixture was heated to 105 °C for 1 h until a brownish-red aqueous solution was obtained. 3-bromopropionic acid (8.70 g, 80 mmol) was dissolved in 20 mL of water and added dropwise to the brownish-red solution, with stirring overnight at room temperature under nitrogen. The reaction mixture was filtered and the pH of the supernatant was adjusted to 3–4 using HCl solution. Then the supernatant was extracted thrice with ethyl acetate. The combined organic layers were washed with water, dried with anhydrous magnesium sulfate, filtered, and recrystallized from ethyl acetate to obtain a red-brown solid (7.30 g, 60% yield).

### Synthesis of selenodipropanoic acid

Synthesis of selenodipropanoic acid was similar with 3,3′-diselanediyldipropanoic acid, without adding another quantity of selenium powder. A final red-brown solid was obtained (65% yield).

### Synthesis of PTX–CIT prodrugs

PTX–CIT prodrugs (PTX–S-CIT, PTX–SS-CIT, PTX–Se-CIT, PTX-SeSe-CIT, PTX–C-CIT, PTX–CC-CIT) were synthesized according to our previous study^16^. Diacid (thiodipropionic acid, 3,3’-dithiodipropionic acid, selenodipropanoic acid, 3,3′-diselanediyldipropanoic acid, pimelic acid or suberic acid, 4 mmol) dissolved in 4 mL acetic anhydride was added to a 50 mL round-bottom flask. At room temperature, the mixture was stirred for 2 h in nitrogen atmosphere. Thereafter, 20 mL methylbenzene was repeatedly added to the system and then dried under reduced pressure for three times. The crude product (anhydride) without further purification was immediately dissolved in 30 mL anhydrous dichloromethane, and then CIT (4 mmol) and DMAP (0.4 mmol) were added. The reaction was stirred at room temperature for 1 h and the process was monitored by thin-layer chromatography (TLC). The crude product was purified by silica gel column chromatography (hexamethylene/ethyl acetate = 5:1, 1‰ glacial acetic acid) to obtain the intermediate products (60% yield). Then, the intermediate product (1 mmol), EDCI (1.5 mmol), HOBt (1 mmol) and DMAP (1 mmol) were dissolved in 50 mL anhydrous dichloromethane and stirred in ice-bath for 2 h under nitrogen atmosphere. After that PTX (1 mmol) was added and the mixture was further stirred at room temperature for 24 h under nitrogen atmosphere. The completion of reaction was monitored by TLC. The target products were purified by preparative liquid chromatography using acetonitrile as mobile phase (65% yield). The prodrugs were confirmed by mass spectrometry (Agilent 1100 Series LC/MSD Trap) and nuclear magnetic resonance spectroscopy (400 MHz ^1^H NMR, Bruker AV-400). The purity of the prodrugs was measured by high performance liquid chromatography (HPLC).

### Preparation and characterization of prodrug nanoassemblies

Prodrug nanoassemblies were prepared by one-step nanoprecipitation method. For non-PEGylated prodrug nanoassemblies, PTX–CIT prodrugs were dissolved in ethanol and then added dropwise into deionized water under stirring. Self-assembly of nanoassemblies occurred spontaneously. For PEGylated prodrug nanoassemblies, PTX–CIT prodrugs and DSPE-PEG_2K_ (20% of total weight) were dissolved in ethanol and then added dropwise into deionized water under stirring. Ethanol in the nano-formulation was then removed under vacuum at room temperature. To prepare dye-labeled prodrug nanoassemblies, coumarin-6 was co-assembled with prodrugs by injecting the mixture of prodrugs, coumarin-6 and DSPE-PEG_2K_ in ethanol into water. The free coumarin-6 was removed by dialysis in pure water. The particle size, polydispersity index (PDI) and zeta potential of prodrug nanoassemblies were measured by Nano ZS Zetasizer instrument (Malvern, UK). Prodrug nanoassemblies solution (5 μL, 1 μmol mL^−1^) was dropped on a carbon-coated copper grid for 30 s and then the samples were stained with 2% phosphotungstic acid (5 μL) for 30 s. The morphology of prodrug nanoassemblies was observed using JEM-2100 transmission electron microscope (TEM, JEOL, Japan).

### Colloidal stability and molecular dynamics simulations

To investigate the colloidal stability of the prepared prodrug nanoassemblies, 1 mL of nano-formulation (1 μmol) was added to 20 mL PBS (pH 7.4, containing 10% of fetal bovine serum (FBS)). The mixtures were incubated at 37 °C with gentle shaking. At prescriptive intervals (0, 2, 4, 6, 8, 12, 24 h), the particle size was measured by Zetasizer (*n* = 3 for each group).

The bond angles of linkages (–S–, –SS–, –Se–, –SeSe–, –C– and –CC–) in PTX–CIT prodrugs were measured by molecular dynamics (MD) simulations using Materials Studio 2017 (BIOVIA, San Diego, CA). First, the molecular were constructed in a cubic lattice with Amorphous Cell module and verified to be chemically correct. The unit cell dimensions were set automatically. Dreiding force field was used to model the atomic interactions. Then, the molecular dynamics calculation was conducted using Forcite module. Briefly, simulation in the NVT (fixed number of particles (N), fixed volume (V), and fixed temperature (T)) ensembles was conducted at 298.0 K. The Nose thermostat was used to control the temperature. The integration time step was set for 1 fs and the total simulation time was performed for 5 ps. In addition, the MD simulation was conducted in a vacuum condition without any gas phase and solvent.

Molecular docking was used to investigate the molecule interaction during self-assemble process. The structures of prodrugs were created using Marvin sketch software. The 3D structures of prodrugs were built by optimizing containing the structural minimization and the structural dynamics optimization with the Sybyl 6.9.1 software package. All other parameters were maintained at the default values. Complexes between two prodrug molecules were predicted by molecular docking using AutoDock 4.0 software. The optimized AutoDocking parameters were as follows: the maximum number of energy evaluations was 25,000,000 per run; the iterations of Solis and Wets local search were 3000; the number of generations was 100, and the number of individuals in population was 300.

### In vitro drug release

The release profiles of PTX from prodrug nanoassemblies were studied using high glucose DMEM medium (containing 30% ethanol (v v^−1^), 10% FBS (v v^−1^), penicillin (100 units mL^−1^) and streptomycin (100 μg mL^−1^)) as release media. The nano-formulation (200 nmol, 1 mL) were incubated in 30 mL release media in the presence of H_2_O_2_ or DTT at 37 °C. At the predetermined time points, the concentration of the released PTX from prodrug nanoassemblies was determined by HPLC (*n* = 3 for each group). The molecular structure of the released PTX was confirmed by high resolution mass spectrum. The release rate was calculated by dividing the amount of released PTX by the amount of PTX at initial time:

1 $${\rm{Cumulative}}\;{\rm{release}}\;{\rm{rate}}\left( \% \right) = C_t\times V/M\times 100$$

*C*_*t*_ is the concentration of PTX at time t, which is measured by HPLC; *V* is the volume of the release media; *M* is the mount of PTX in prodrug nanoassemblies at initial time.

### Cell culture

KB cells, A549 cells and 4T1 cells were obtained from COBIOER Biotechnology Co., Ltd (Nanjing, China), and all these cells were tested negative for mycoplasma contamination. KB cells and A549 cells were authenticated by Short Tandem Repeat (STR) profiling. KB cells are contaminated by Hela cells, and are listed in the database of commonly misidentified cell lines by International Cell Line Authentication Committee (ICLAC). In this study, KB cells were just used to evaluated the in vitro cytotoxicity and in vivo antitumor efficiency of prodrug nanoassemblies. HeLa cells are also widely used to evaluated the cytotoxicity and antitumor efficiency of chemotherapeutics drugs, and Hela cells are also very sensitive to PTX. Therefore, being used as an evaluation model instead of a disease model, the potential contamination of KB cells by Hela cells might not significantly influence the pharmacodynamic evaluation of prodrug nanoassemblies under the same experimental conditions. Moreover, the in vitro cytotoxicity of prodrug nanoassemblies were further validated using A549 cells and 4T1 cells, and the in vivo antitumor efficiency of prodrug nanoassemblies were further investigated using a 4T1 tumor-bearing BALB/c mice.

KB cells and A549 cells were cultured in high glucose DMEM medium with 10% FBS, penicillin (100 units mL^−1^) and streptomycin (100 μg mL^−1^). 4T1 cells were cultured in Gibico RPMI 1640 medium with 10% FBS, penicillin (100 units mL^−1^) and streptomycin (100 μg mL^−1^). All cells were maintained in a humidified atmosphere of 5% CO_2_ at 37 °C.

### Cytotoxicity assay

The antiproliferative activities of prodrug nanoassemblies against KB, A549 and 4T1 cells were evaluated by MTT assay. Briefly, cells were seeded into 96-well plates at a density of 3000 cells per well and incubated for 24 h. Then, cells were treated with serial dilutions of DSPE-PEG_2K_, Taxol or prodrug nanoassemblies and further incubated for 48 or 72 h. The cells without any treatment were utilized as control (*n* = 3 for each group). After incubation, 50 μL of MTT solution (5 mg mL^−1^) was added and treated for another 4 h. Then 200 μL DMSO was added, and the absorbance at 570 nm was detected by a microplate reader (Model 500, USA). The IC_50_ values were calculated by IBM SPSS Statistic 20, using molar concentration and inhibition ratio as parameters.

### Intracellular drug release

KB cells were seeded into 24-well plates at a density of 1 × 10^5^ cells per well and incubated for 24 h. Then Taxol and prodrug nanoassemblies (drug concentrations: 58.6, 117.1 and 234.2 nmol L^−1^) were added and incubated for 48 h. After that, the cells together with the culture media were collected. After sonication and centrifugation, the concentrations of free PTX in the supernatants were measured by UPLC-MS-MS (ACQUITY UPLCTM, Waters Co., Ltd., Milford, MA, USA).

### Intracellular ROS and GSH level

DCFH-DA was used to measure the intracellular ROS level. Briefly, KB cells were seeded into 12-well plates at a density of 1 × 10^5^ cells per well and incubated for 24 h. Then Taxol and prodrug nanoassemblies (drug concentrations: 50 and 100 nmol L^−1^) were added and incubated for 12 h. The positive control group was incubated with blank culture medium for 12 h and then treated with Rosup (Beyotime Biotechnology, China) for 30 min. After that, the cells were treated with DCFH-DA for 30 min. Intracellular fluorescence intensity was measured by FACS calibur flow cytometer.

To measure the intracellular concentration of GSH and GSSG, KB cells were seeded into 6-well plates at a density of 2 × 10^5^ cells per well and incubated for 24 h. Then Taxol and prodrug nanoassemblies (drug concentrations: 50 and 100 nmol L^−1^) were added and incubated for 12 h. The intracellular concentration of GSH and GSSG was measured using a GSH and GSSG Assay Kit (Beyotime Biotechnology, China).

### Cellular uptake

KB cells were used to evaluate the cellular uptake of prodrug nanoassemblies. Briefly, KB cells were seeded in 24-well plates at a density of 1 × 10^4^ cells per well and incubated for 24 h. Then, cells were washed and incubated with free coumarin-6 solution or coumarin-6-labeled prodrug nanoassemblies (10 μmol L^−1^) with equivalent concentration of coumarin-6 (250 ng mL^−1^) for 0.5 h or 2 h at 37 °C. Then, cells were washed with cold PBS for three times and fixed with 4% formaldehyde for 10 min at room temperature. After that the cells were washed again and the nuclei were counterstained by Hoechst for 10 min. The prepared covered slips were examined by confocal laser scanning microscopy (CLSM, TCS SP2/AOBS, LEICA, Germany).

For quantitative determination, KB cells were seeded in 12-well plates at a density of 1 × 10^5^ cells per well for 24 h. Then, the medium was replaced with free coumarin-6 solution or coumarin-6-labeled prodrug nanoassemblies (20 μmol L^−1^) with equivalent concentration of coumarin-6 (500 ng mL^−1^) for 0.5 h or 2 h at 37 °C. After that, the cells were washed, collected and re-suspended in PBS. Intracellular fluorescence intensity was measured by FACS calibur flow cytometer.

### Animal studies

All the animals were obtained from the Laboratory Animal Center of Shenyang Pharmaceutical University (Shenyang, Liaoning, China). All the animal experiments were conducted according to the Guidelines for the Care and Use of Laboratory Animals, and received ethical approval from the Institutional Animal Ethical Care Committee (IAEC) of Shenyang Pharmaceutical University.

### In vivo pharmacokinetic study

Male Sprague-Dawley rats (200–250 g) were used to evaluate the pharmacokinetic profiles of prodrug nanoassemblies. Prior to the experiments, the rats were fasted for 12 h with free access to water. The animals were intravenously administrated with Taxol or prodrug nanoassemblies at a dose of 2.9 μmol kg^−1^ (*n* = 5 for each group). At the predetermined time points (0.03, 0.17, 0.5, 1, 2, 3, 4, 8, 12 and 24 h), blood samples were collected and then centrifuged to obtain the plasma. The concentration of prodrugs and PTX were measured by UPLC-MS-MS. AUC, t_1/2_ and MRT were calculated using DAS 2.0 (Shanghai BioGuider Medicinal Technology Co. Ltd., China).

### In vivo biodistribution

4T1 tumor-bearing BALB/c mice were utilized to investigate the biodistribution of prodrug nanoassemblies. When the tumor volume reached approximately 400 mm^3^, Taxol and prodrug nanoassemblies were intravenously administrated at a dose of 9.3 μmol kg^−1^ (*n* = 3 for each group). After 1, 4 and 12 h post injection, the mice were killed and the major organs (heart, liver, spleen, lung and kidney) and tumors were collected. The concentration of prodrugs and PTX were measured by UPLC-MS-MS.

### In vivo antitumor efficacy

KB cells (5 × 10^6^ cells per 100 μL) were injected subcutaneously into the flank of female nude mice. When tumor volume reached approximately 150 mm^3^, mice were intravenously administrated with Taxol or prodrug nanoassemblies at a dose of 9.3 μmol kg^−1^ (*n* = 5 for each group). Saline was used as control. Preparations were administrated every other day for a total of five injections. Body weight and tumor volume were monitored every day. After 3 days of the last treatment, mice were sacrificed, and blood was collected for hepatic and renal function analysis. The major organs (heart, liver, spleen, lung, and kidney) and tumor tissues were collected and fixed by formalin for hematoxylin and eosin (H&E) (Wanleibio. China). The apoptosis of tumor cell was evaluated by TUNEL assay using an apoptosis detection kit. Nucleus was stained by DAPI, and the processed slides were observed using an Olympus IX71 inverted microscope (Japan). Ki-67 immunofluorescence staining was used to investigate the proliferation of tumor cells. After dewaxing and rehydrating, the tumor sections were immersed into pH 6.0 citrate buffer (95 ˚C, 10 min) for antigen retrieval. Then, tumor sections were blocked with 10% goat serum for 20 min and incubated with Ki-67 primary antibody (ABclonal, China, diluted for 100 times) for 12 h at 4 °C. After that, biotin-labeled second antibody (BOSTER biological technology, China, diluted for 200 times) was added and incubated for 30 min at 37 °C. Finally, tumor sections were incubated with SABC-FITC (BOSTER biological technology, China, diluted for 200 times) for 30 min at 37 °C, and the nucleus was stained by DAPI. The processed slides were observed using CLSM.

The in vivo antitumor efficiency of prodrug nanoassemblies was also investigated on 4T1 tumor-bearing BALB/c mice using the same experimental scheme.

2 $${\rm{Tumor}}\;{\rm{volume}}\left( {{\rm{mm}}^3} \right) = {\it{L}}\times {\it{W}}\times {\it{W}}/2$$

*L* is the long diameter of tumor; *W* is the short diameter of tumor.

3 $${\rm{Tumor}}\;{\rm{burden}}\left( \% \right) = {\it{W}}_{{\it{{\rm{tumor}}}}}/{\it{W}}_{{\it{{\rm{mice}}}}}\times 100$$

*W*_tumor_ is the weight of tumor, *W*_mice_ is the weight of mice.

### Statistical analysis

Data were calculated and processed as mean ± SD. Comparison between groups was analyzed with student’s *T* test (two-tailed) and one-way analysis of variance (ANOVA). Statistical differences were considered significant at *P* < 0.05.

### Reporting Summary

Further information on research design is available in the Nature Research Reporting Summary linked to this article.

## Supplementary information

Supplementary Information

Reporting Summary

## Source data

source data

## Data Availability

All relevant data are available from the authors. The source data underlying Figs. 2, 3a–c, 4, 5a, b, 5d, e, as well as Supplementary Figures 9, 18–21, 23, 25, 27, 28, 32, Supplementary Table 1 and Supplementary Table 2 are provided as a Source Data file.
