# Supplementary material for: Probing the impact of sulfur/selenium/carbon linkages on prodrug nanoassemblies for cancer therapy
Source: Nat Commun. 2019 Jul 19;10:3211. doi: 10.1038/s41467-019-11193-x (PMC6642185; doi:10.1038/s41467-019-11193-x)
Supplement: Supplementary file 1 — Supplementary Information [file 41467_2019_11193_MOESM1_ESM.pdf]

Supplementary Information

Probing the impact of sulfur/selenium/carbon linkages on prodrug nanoassemblies for cancer therapy

Sun et al.

Supplementary Figures

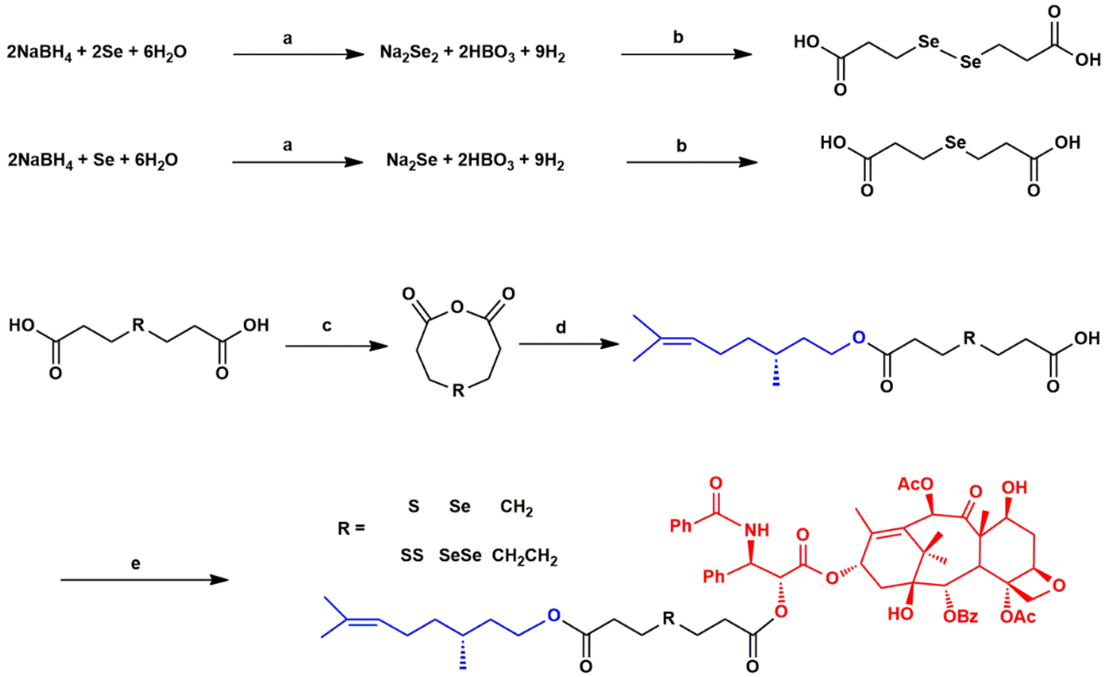

Supplementary Figure 1. The synthetic route of PTX-CIT prodrugs. (a) 0 °C, 30 min; 105 °C, 1 h; (b) 25 °C, 12 h; (c) acetic anhydride, 25 °C, 2 h; (d) CIT, DMAP, 25 °C, 1 h; (e) EDCI, HOBt, DMAP, 0 °C, 2 h; PTX, 25 °C, 24 h.

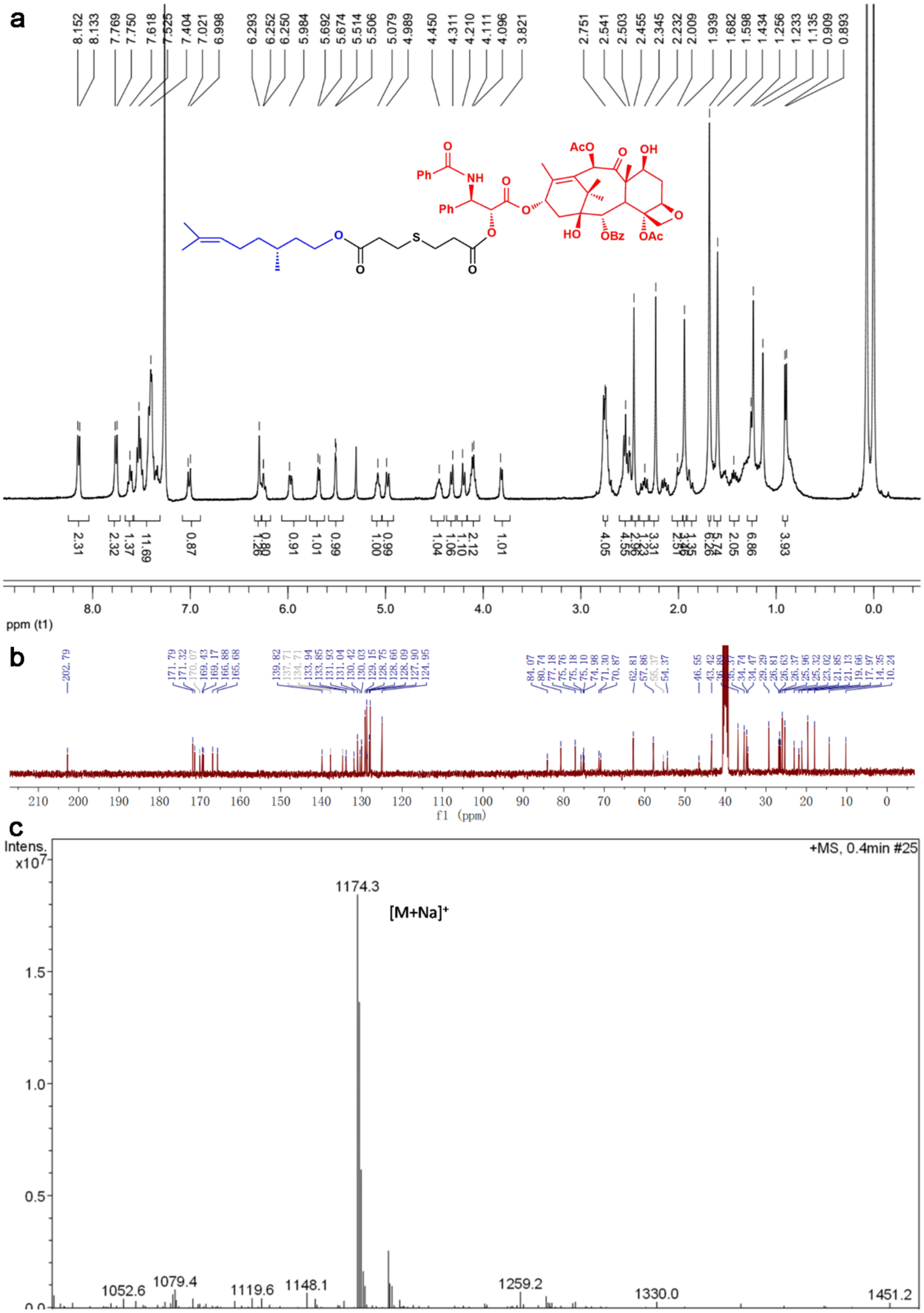

Supplementary Figure 2. Structure confirmation of PTX-S-CIT. (a) ^{1}H NMR. (b) ^{13}C NMR. (c) MS.

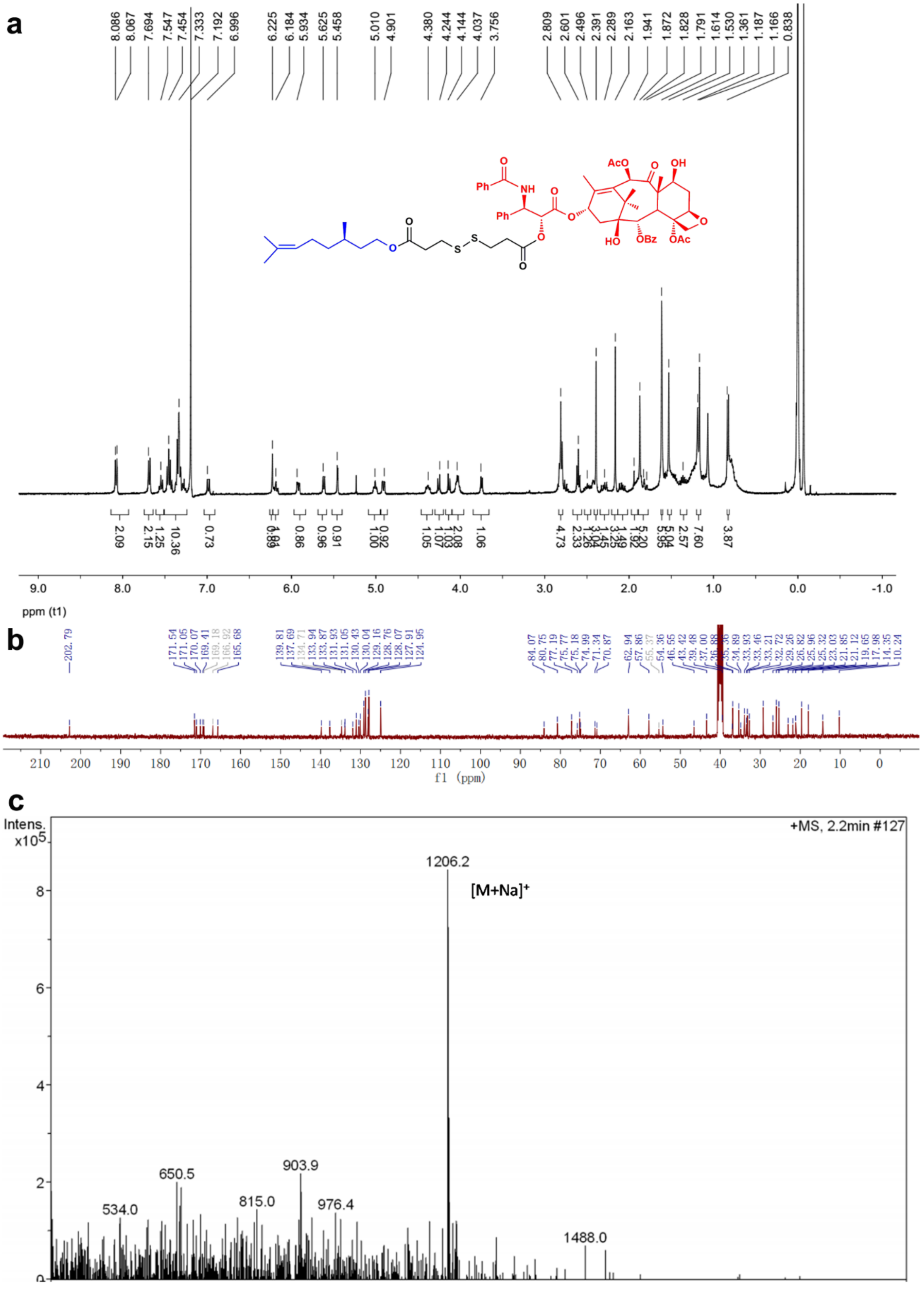

Supplementary Figure 3. Structure confirmation of PTX-SS-CIT. (a) ^{1}H NMR. (b) ^{13}C NMR. (c) MS.

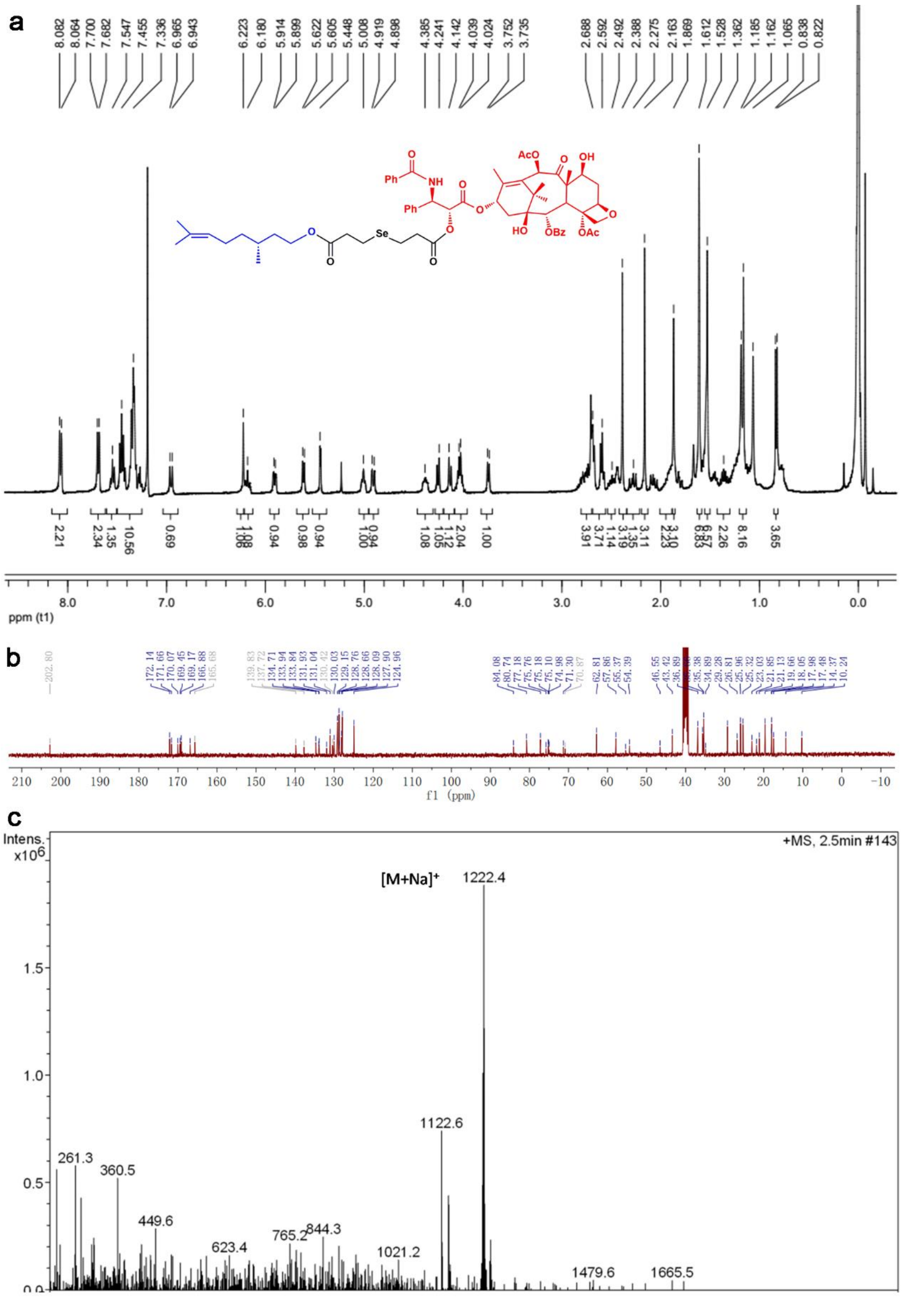

Supplementary Figure 4. Structure confirmation of PTX-Se-CIT. (a) ^{1}H NMR. (b) ^{13}C NMR. (c) MS.

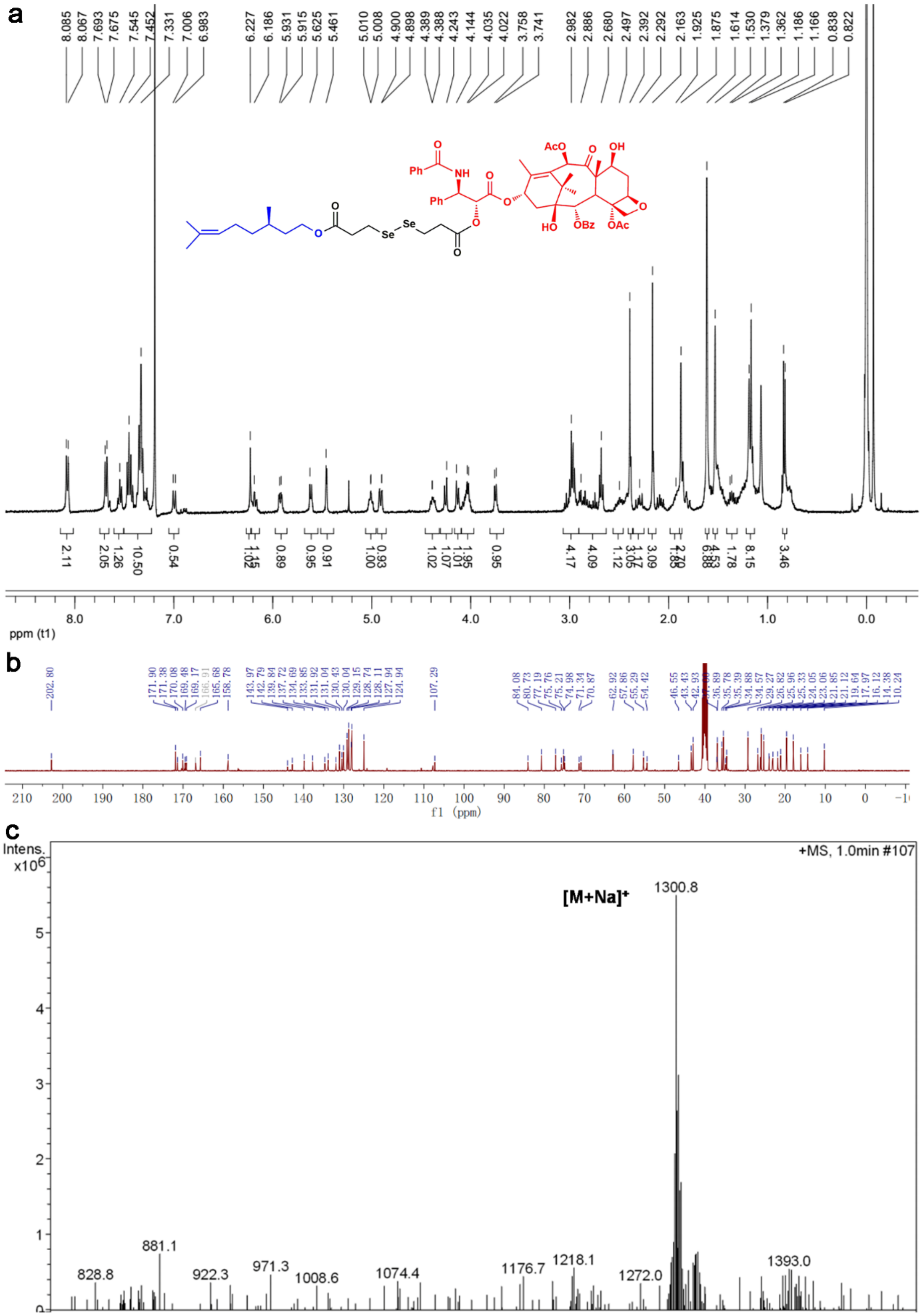

Supplementary Figure 5. Structure confirmation of PTX-SeSe-CIT. (a) ^{1}H NMR. (b) ^{13}C NMR. (c) MS.

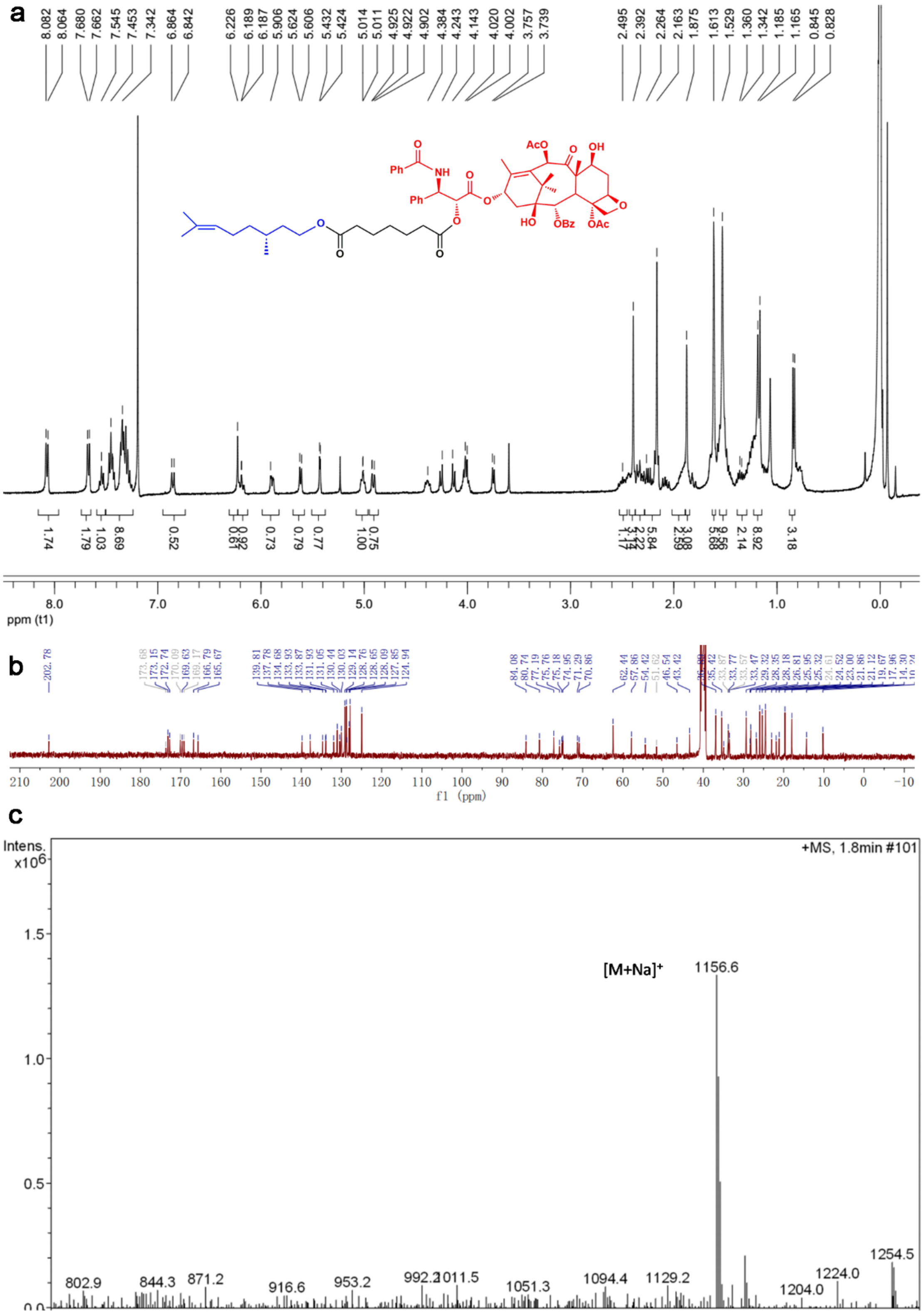

Supplementary Figure 6. Structure confirmation of PTX-C-CIT. (a) ^{1}H NMR. (b) ^{13}C NMR. (c) MS.

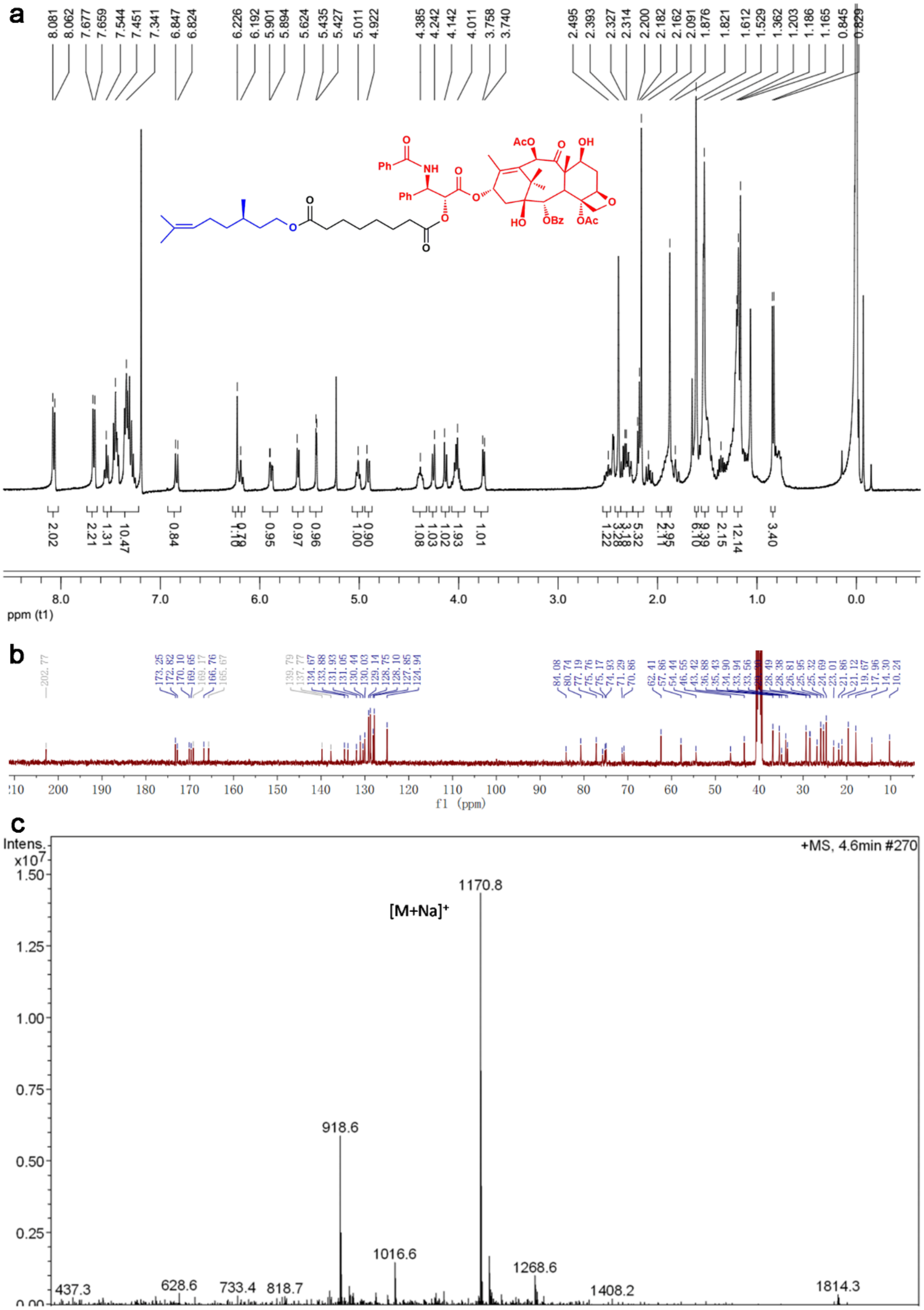

Supplementary Figure 7. Structure confirmation of PTX-CC-CIT. (a) ^{1}H NMR. (b) ^{13}C NMR. (c) MS.

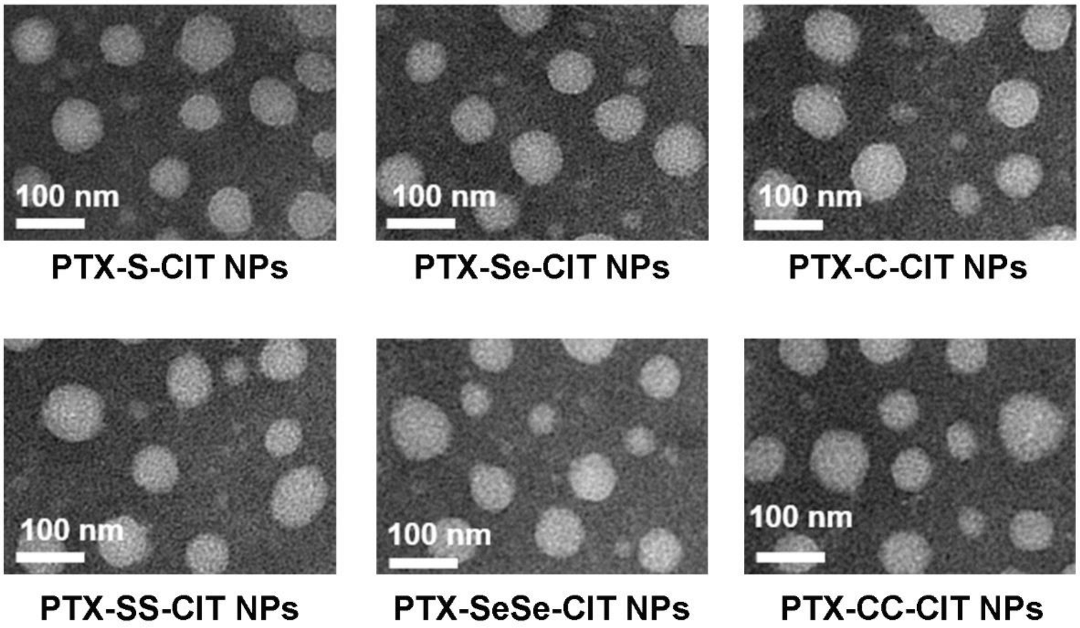

Supplementary Figure 8. Morphology characteristic. TEM images of PTX-CIT prodrug nanoassemblies.

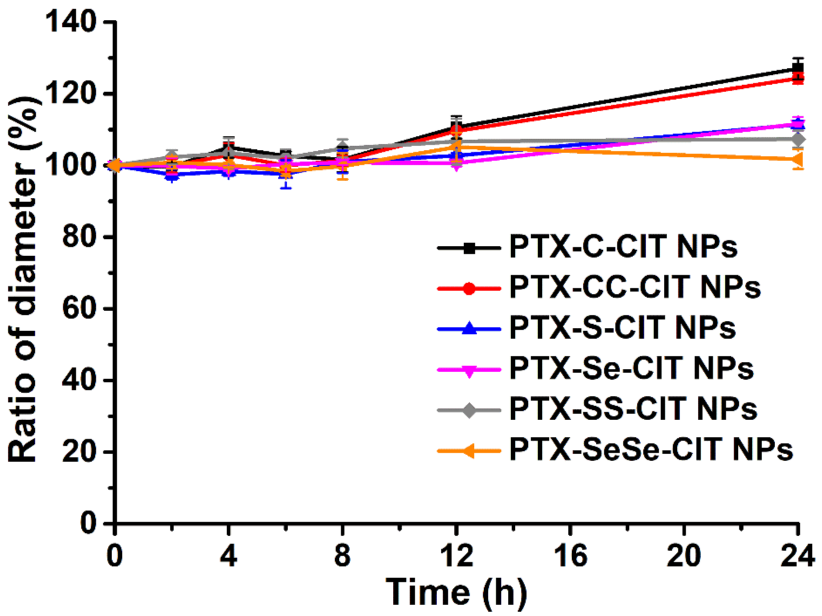

Supplementary Figure 9. The colloidal stability of prodrugs nanoassemblies. The changes of particle size of prodrugs nanoassemblies in pH 7.4 PBS supplemented with 10% FBS at 37 °C. Data are presented as mean ± SD (three independent experiments).

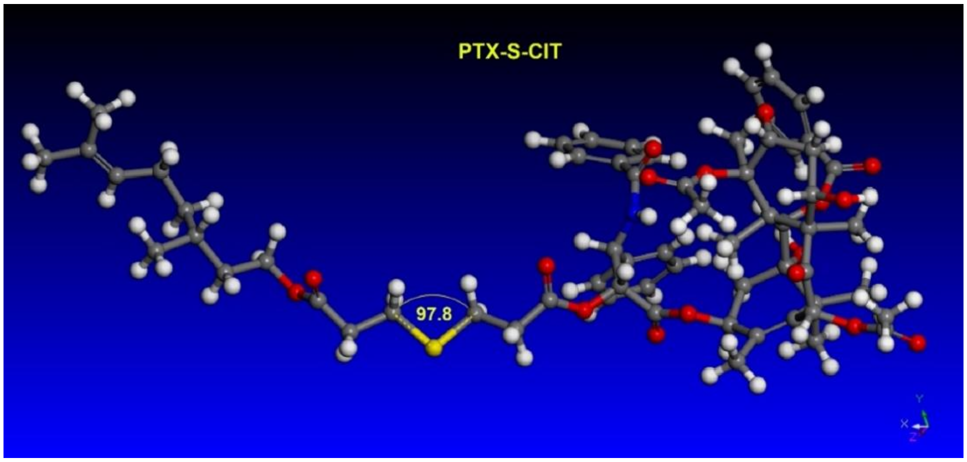

Supplementary Figure 10. Molecular simulation. The bond angle of -S- in PTX-S-CIT prodrug.

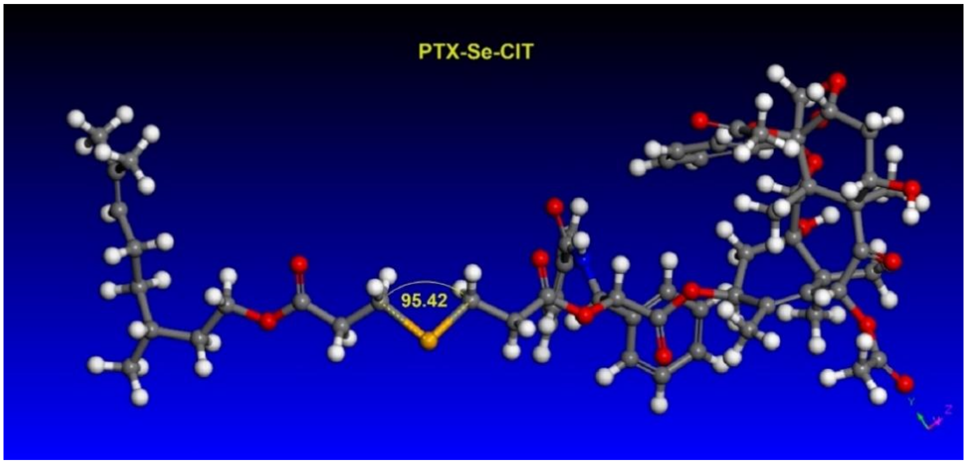

Supplementary Figure 11. Molecular simulation. The bond angle of -Se- in PTX-Se-CIT prodrug.

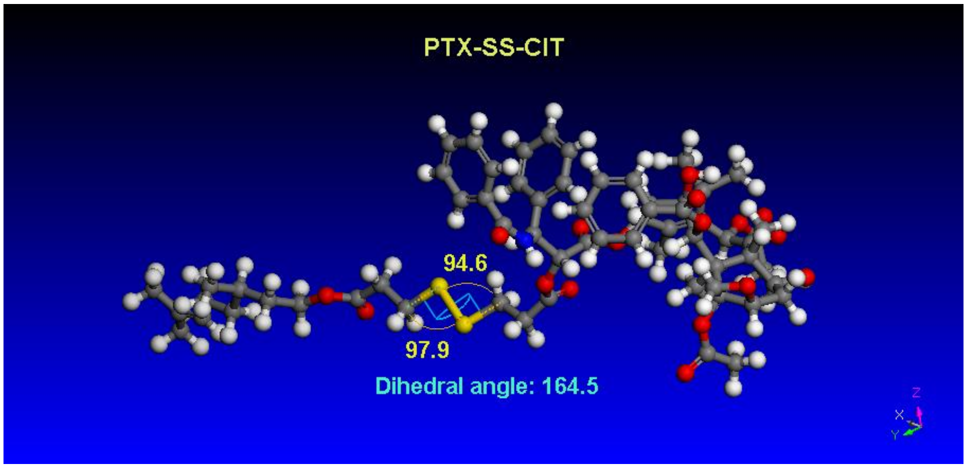

Supplementary Figure 12. Molecular simulation. The bond angle and dihedral angle of -SS- in PTX-SS-CIT prodrug.

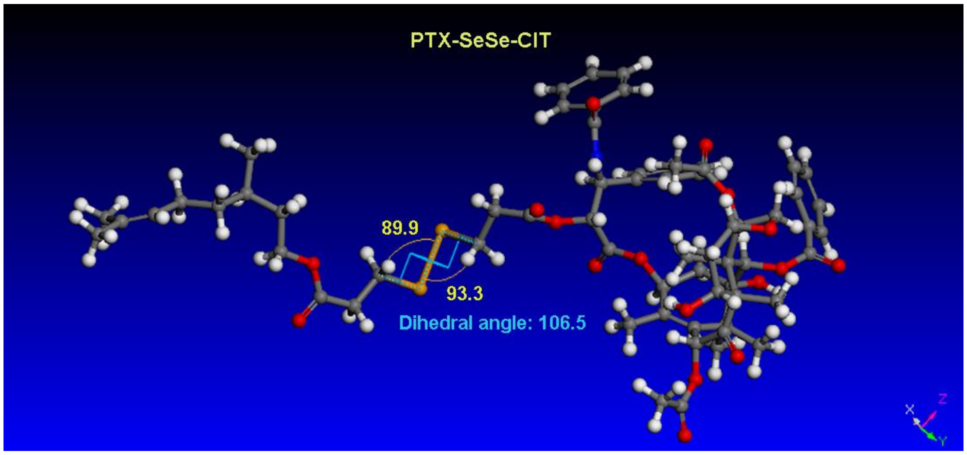

Supplementary Figure 13. Molecular simulation. The bond angle and dihedral angle of -SeSe- in PTX-SeSe-CIT prodrug.

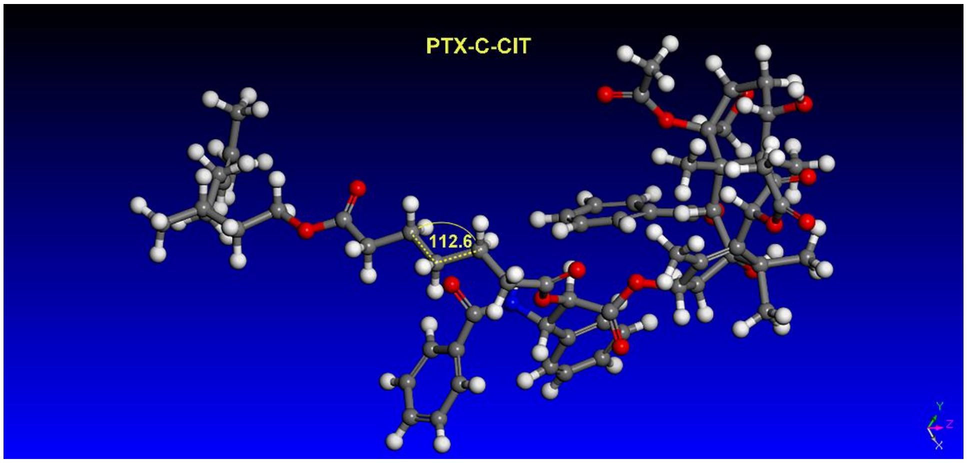

Supplementary Figure 14. Molecular simulation. The bond angle of -C- in PTX-C-CIT prodrug.

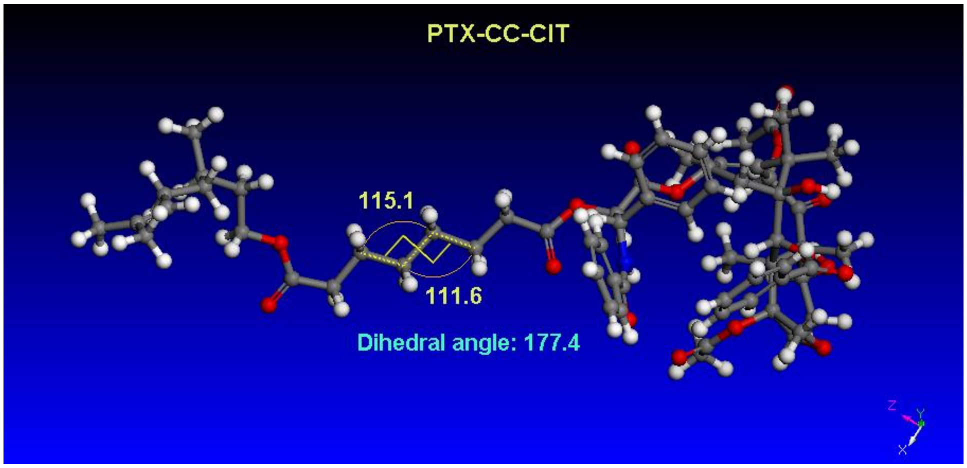

Supplementary Figure 15. Molecular simulation. The bond angle and dihedral angle of -CC- in PTX-CC-CIT prodrug.

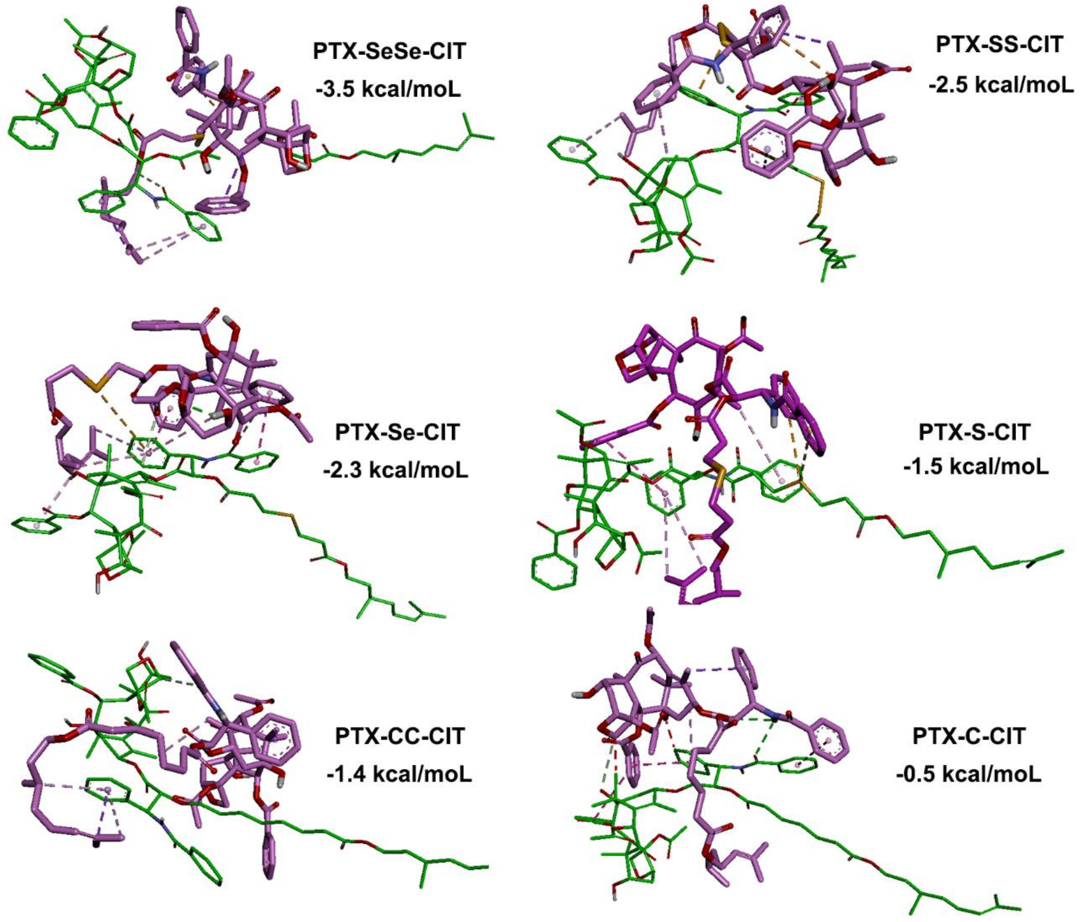

Supplementary Figure 16. Molecular docking. The binding energy of two prodrug molecules.

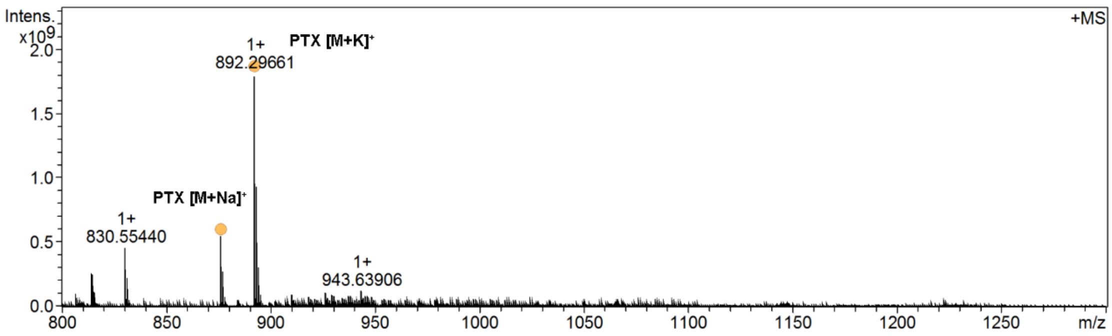

Meas. m/z	#	Ion Formula	m/z
876.323012	1	C47H51NNaO14	876.320176
892.296612	1	C47H51KNO14	892.294113

Supplementary Figure 17. Structure confirmation. Mass spectra of the released PTX. The $[M+Na]^+$ and $[M+K]^+$ ion peaks of PTX were marked.

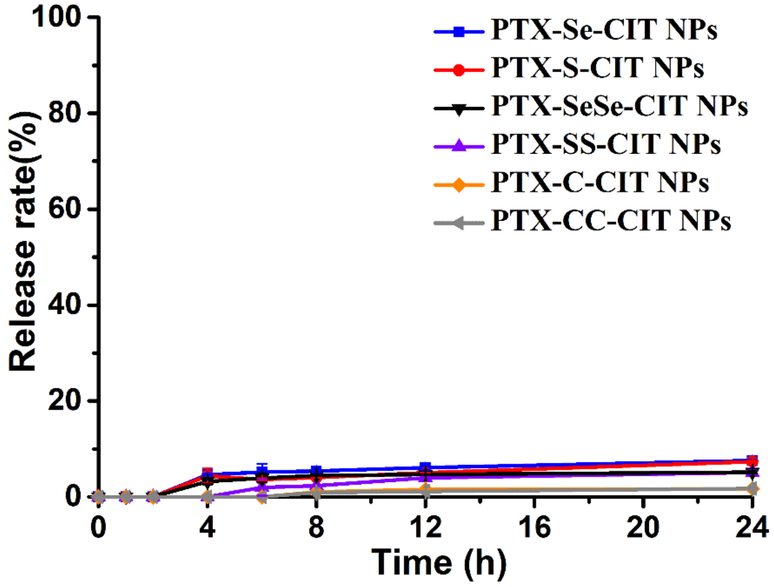

Supplementary Figure 18. *In vitro* drug release of prodrug nanoassemblies. Blank medium without H_2O_2 and DTT was used as release medium. Data are presented as mean ± SD (three independent experiments).

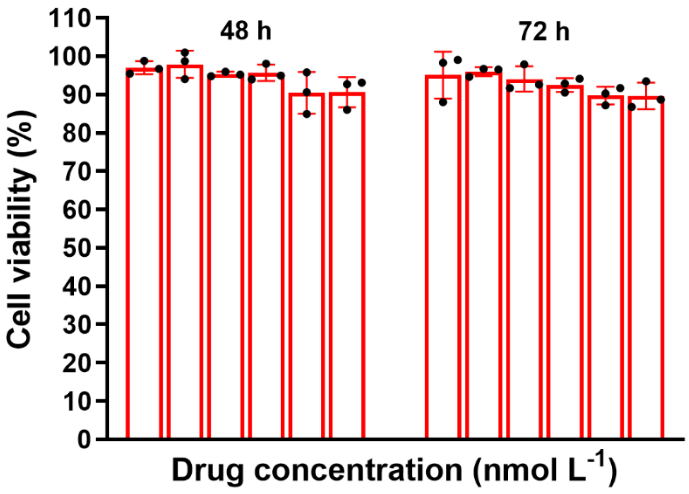

Supplementary Figure 19. Cytotoxicity assay of DSPE-PEG$_{2k}$. Viability of KB cells after treated with various concentrations of DSPE-PEG$_{2k}$ for 48 h and 72 h. Data are presented as mean ± SD (three independent experiments).

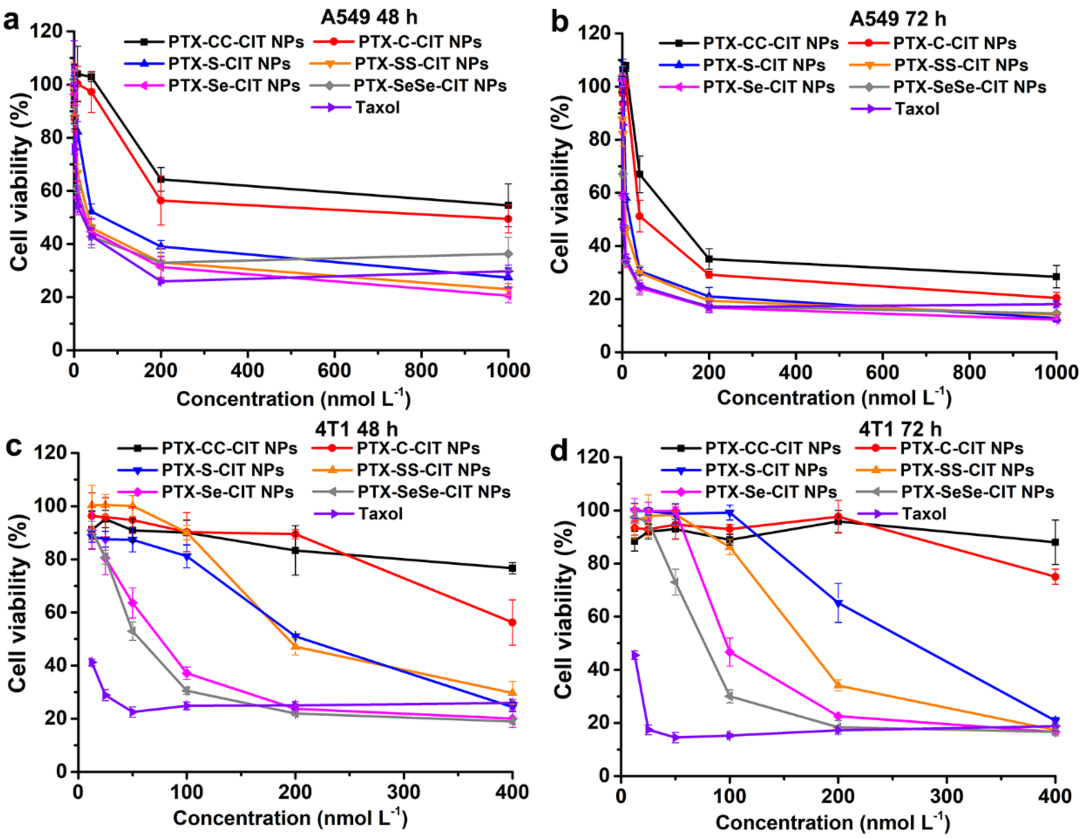

Supplementary Figure 20. Cytotoxicity assay of prodrug nanoassemblies. Viability of A549 cells after treated with various concentrations of Taxol and prodrug nanoassemblies for (a) 48 h and (b) 72 h. Viability of 4T1 cells after treated with various concentrations of Taxol and prodrug nanoassemblies for (c) 48 h and (d) 72 h. Data are presented as mean ± SD (three independent experiments).

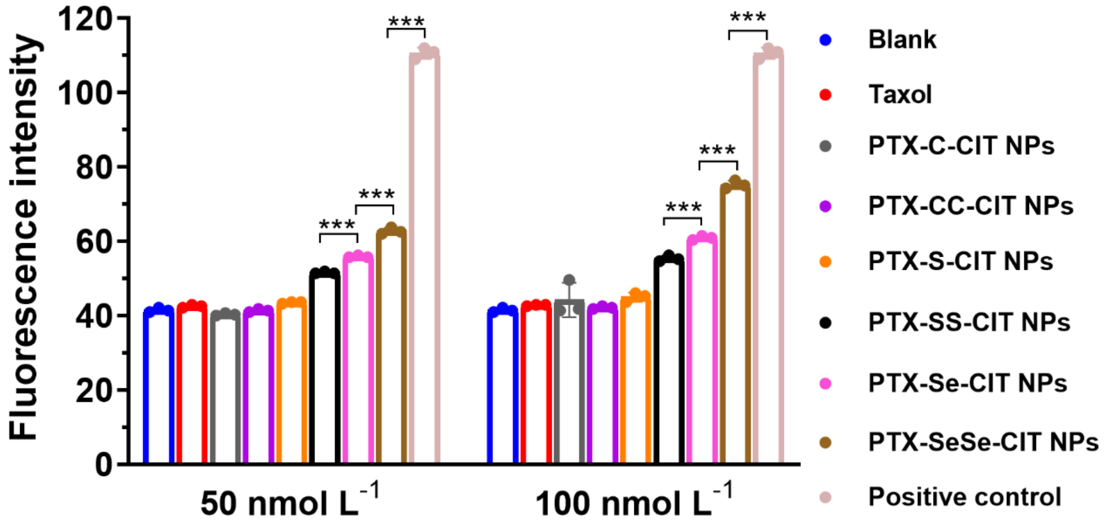

Supplementary Figure 21. Intracellular ROS level. The influence of Taxol and prodrug nanoassemblies on the intracellular ROS level of KB cells after incubation for 12 h. DCFH-DA was used to measure the intracellular ROS level. Data are presented as mean ± SD (three independent experiments). *** $P < 0.001$ by two-tailed Student's t-test.

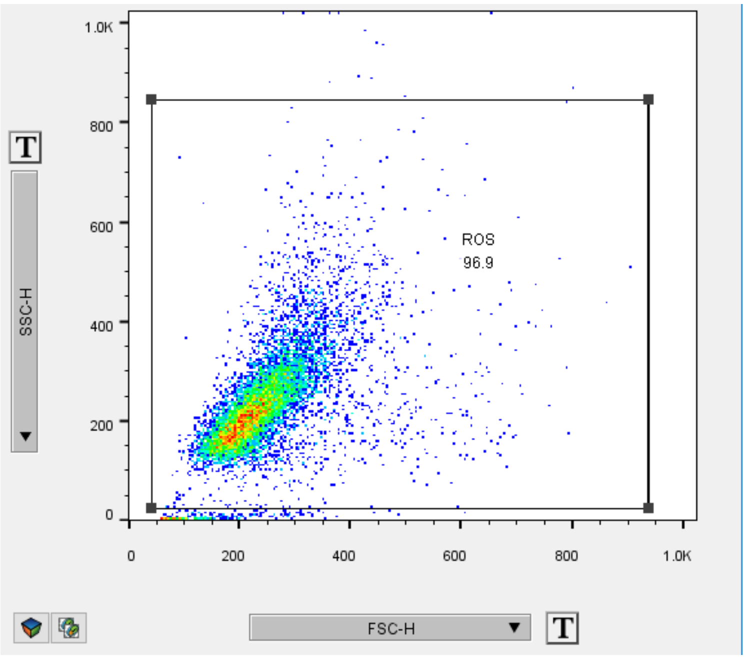

Supplementary Figure 22. Gating strategy. The gating strategy used for cell sorting in the intracellular ROS study presented on Supplementary Figure 21. Rectangle gates based on FSC-H and SSC-H signals was used to exclude cell debris and clumps. DCFH-DA was used as the fluorochrome.

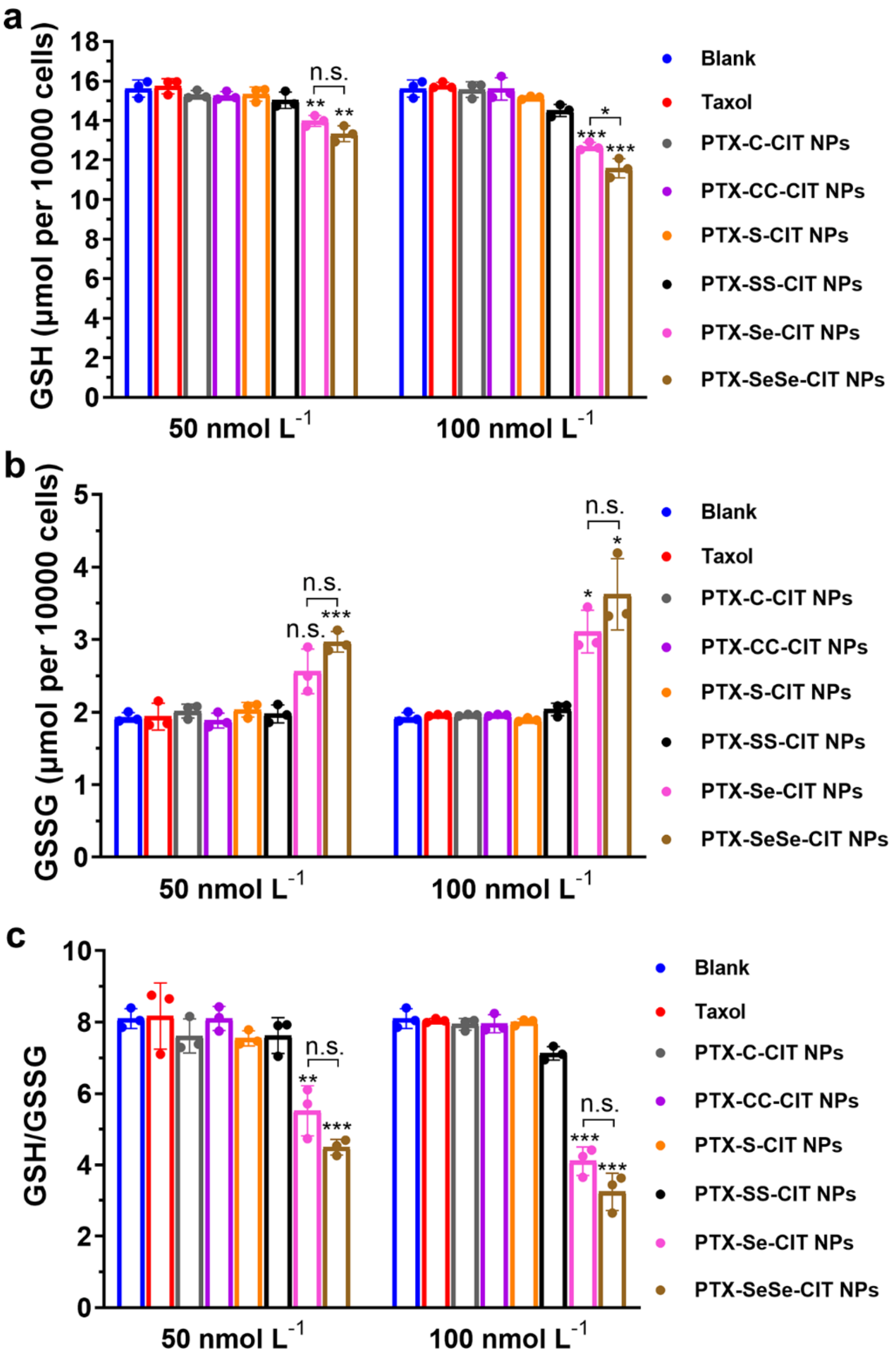

Supplementary Figure 23. Intracellular GSH/GSSG level. The influence of Taxol and prodrug nanoassemblies on the intracellular GSH (a), GSSG (b) and GSH/GSSG ratio (c) of KB cells after incubation for 12 h. Data are presented as mean ± SD (three independent experiments). * $P < 0.05$, ** $P < 0.01$, *** $P < 0.001$ versus blank media as the control (Two-tailed Student's t-test).

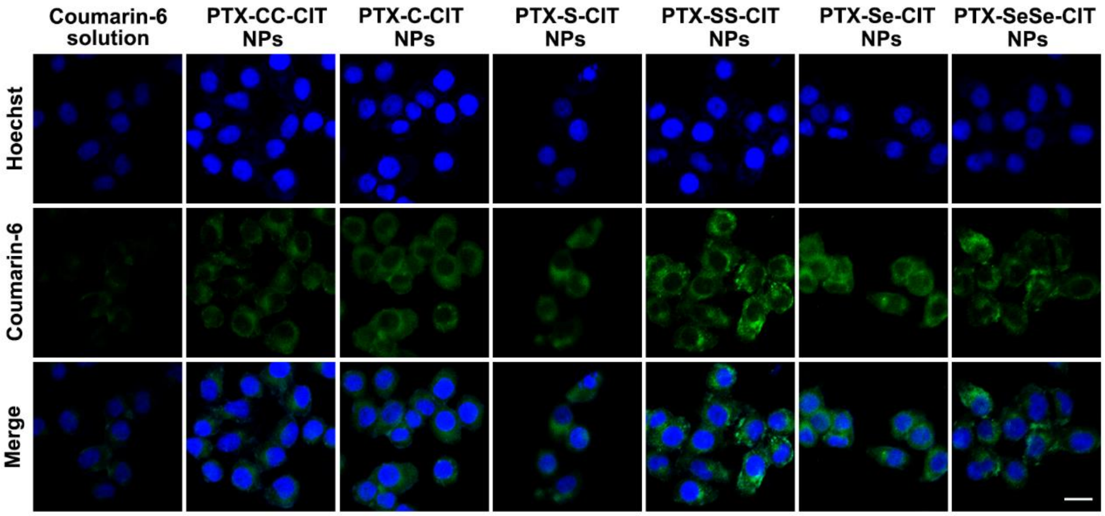

Supplementary Figure 24. Cellular uptake. Confocal laser scanning microscopy (CLSM) images of KB cells incubated with free coumarin-6 or coumarin-6-labeled prodrug nanoassemblies for 0.5 h. Scale bar represents 10 μm.

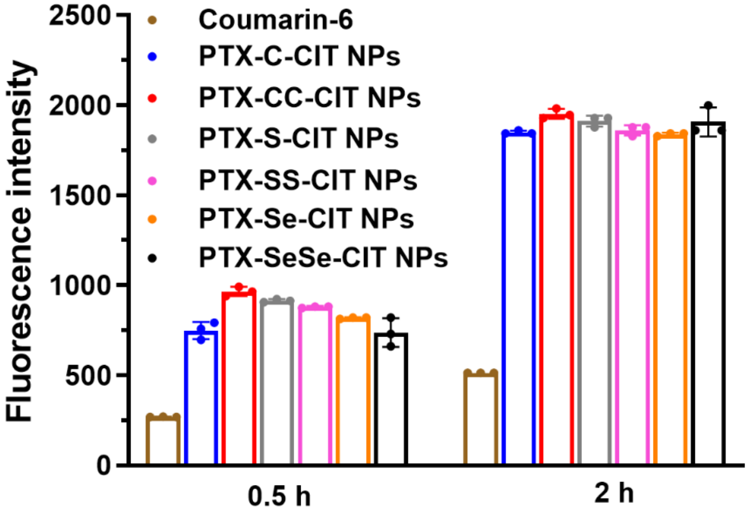

Supplementary Figure 25. Cellular uptake. Cellular uptake of free coumarin-6 (control) and coumarin-6-labeled prodrug nanoassemblies after incubation with KB cells for 0.5 h and 2 h by flow cytometry. Data are presented as mean ± SD (three independent experiments).

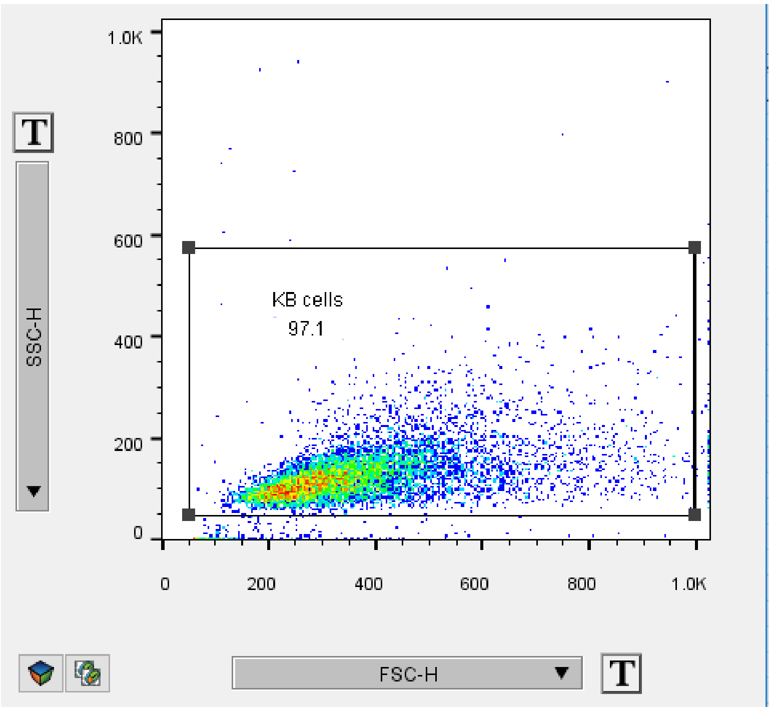

Supplementary Figure 26. Gating strategy. The gating strategy used for cell sorting in the cellular uptake study presented on Supplementary Figure 25. Rectangle gates based on FSC-H and SSC-H signals was used to exclude cell debris and clumps. Coumarin-6 was used as the fluorochrome.

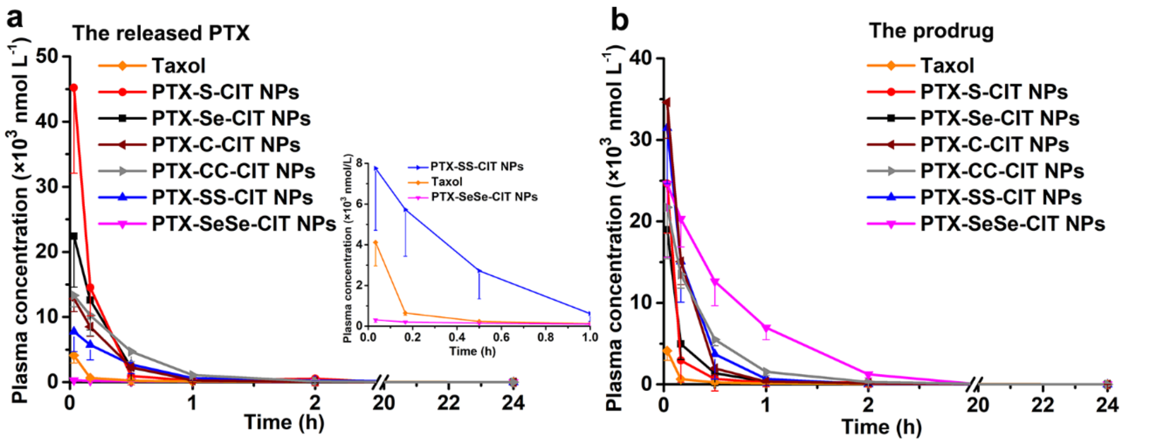

Supplementary Figure 27. Pharmacokinetic profiles of prodrug nanoassemblies. Molar concentration-time curves of the released PTX (a) and the prodrugs (b). Data are presented as mean ± SD (five independent experiments).

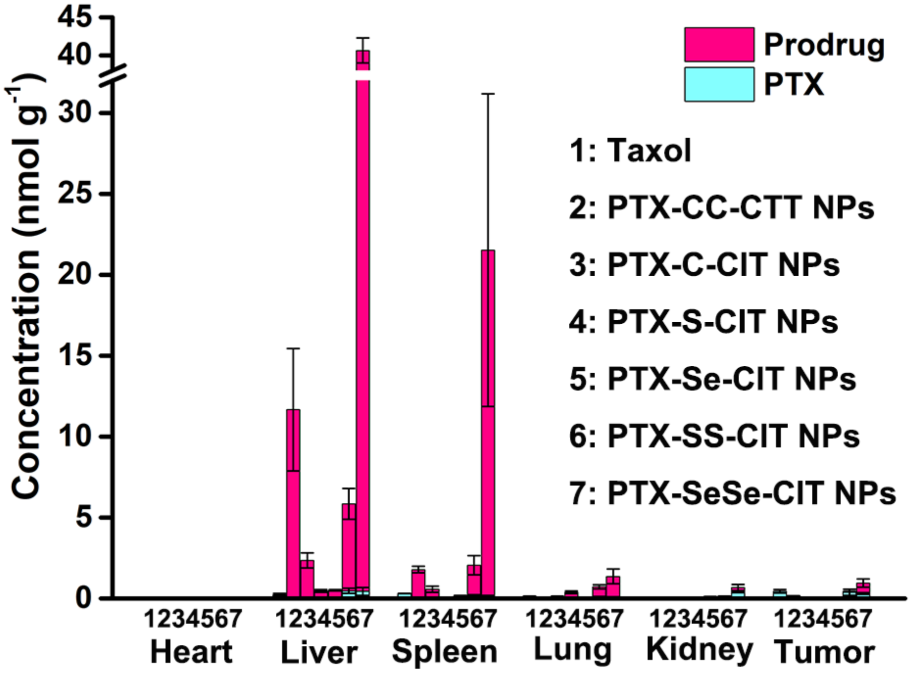

Supplementary Figure 28. Biodistribution. *In vivo* biodistribution of Taxol and prodrug nanoassemblies at 12 h. Data are presented as mean ± SD (three independent experiments).

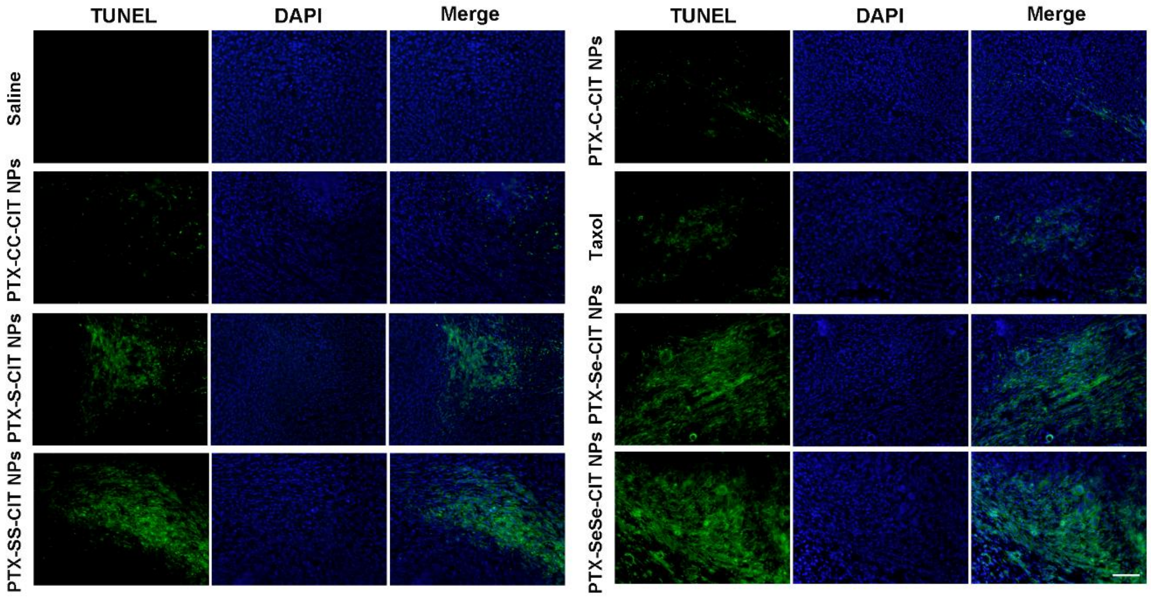

Supplementary Figure 29. TUNEL assay. KB tumor sections were prepared after the last treatment. Scale bar represents 50 μm.

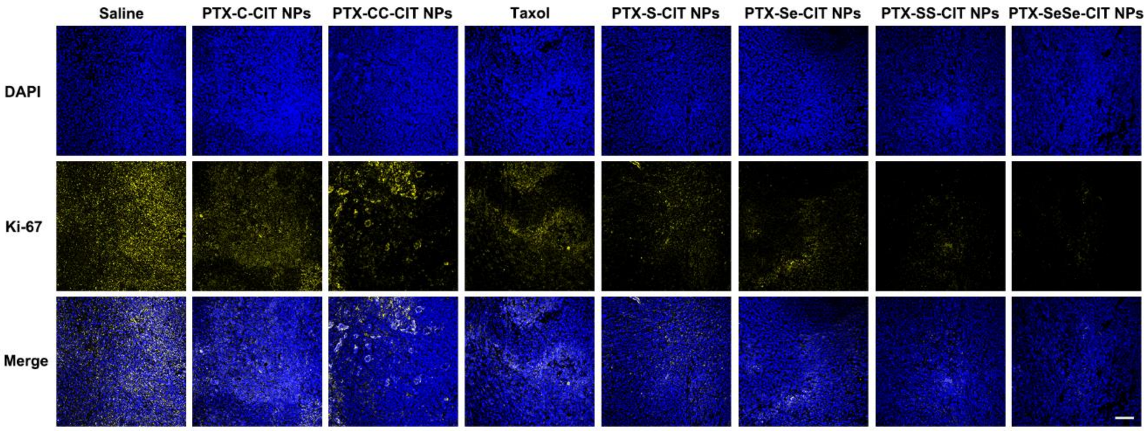

Supplementary Figure 30. Ki-67 immunofluorescence staining. KB tumor sections were prepared after the last treatment. Scale bar represents 50 μm.

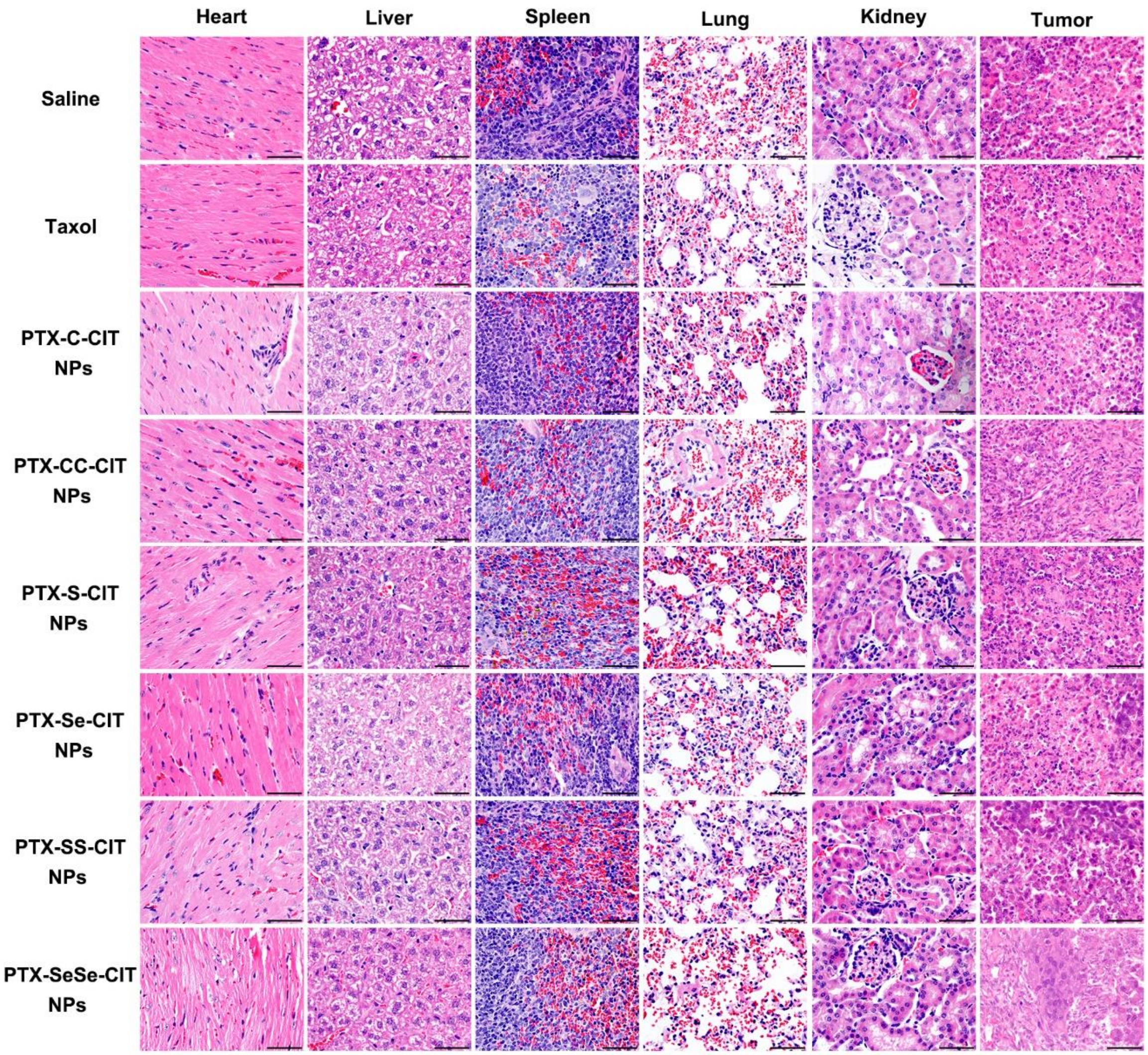

Supplementary Figure 31. H&E staining. The major organs and tumor sections of KB tumor bearing nude mice were prepared after the last treatments. Scale bar represents 50 μm.

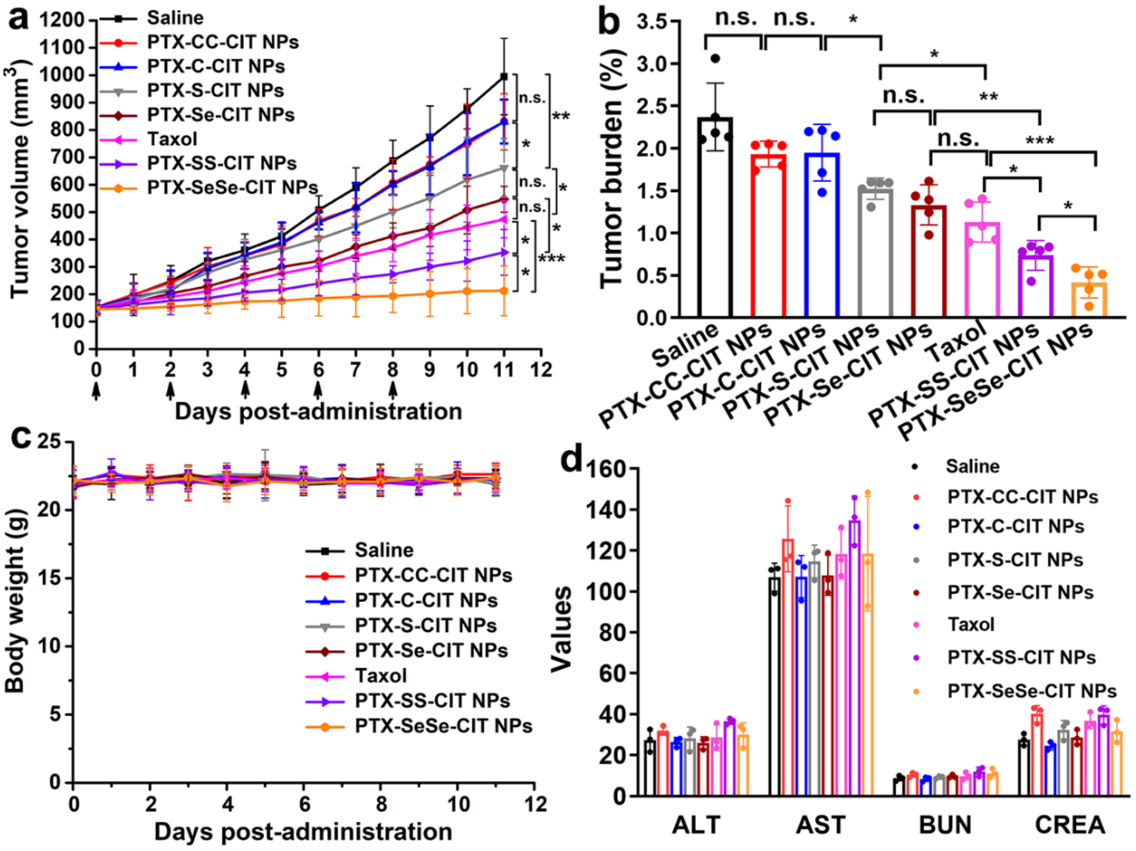

Supplementary Figure 32. *In vivo* antitumor efficacy of prodrug nanoassemblies. 4T1 xenograft tumors was established. (a) Tumor volume. (b) Tumor burden. (c) Body weight changes. Data are presented as mean ± SD (five independent experiments). * $P < 0.05$, ** $P < 0.01$ and *** $P < 0.001$ by two-tailed Student's t-test. (d) Hepatorenal function parameters. Data are presented as mean ± SD (three independent experiments). AST: aspartate aminotransferase (U L^{-1}); ALT: alanine aminotransferase (U L^{-1}); BUN: blood urea nitrogen (mmol L^{-1}); CREA: creatinine (μmol L^{-1}).

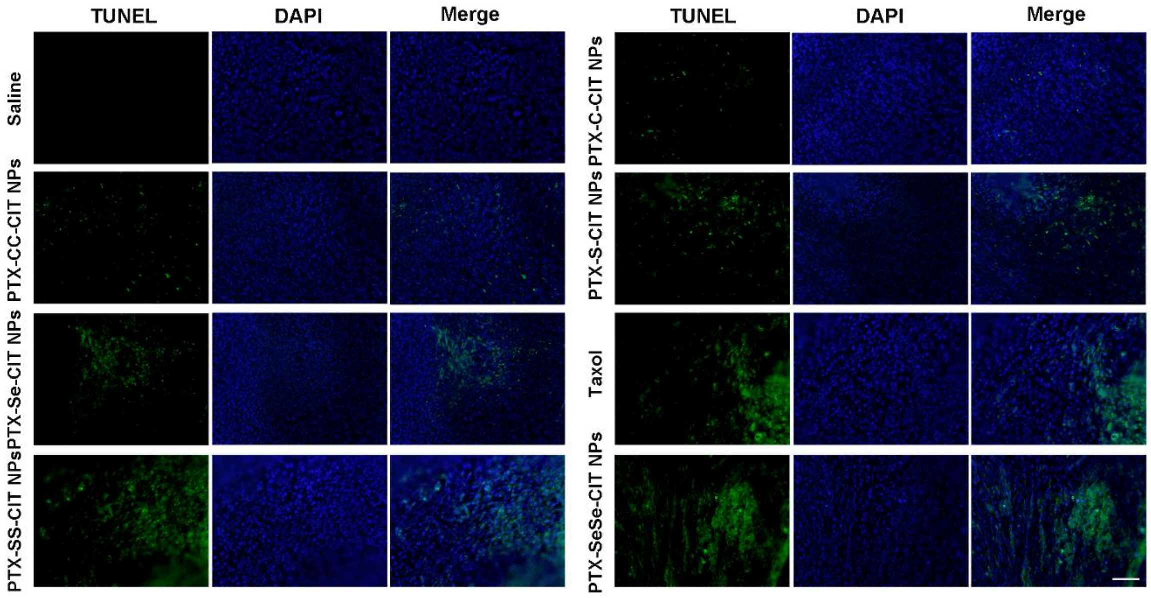

Supplementary Figure 33. TUNEL assay. 4T1 tumor sections were prepared after the last treatment. Scale bar represents 50 μm.

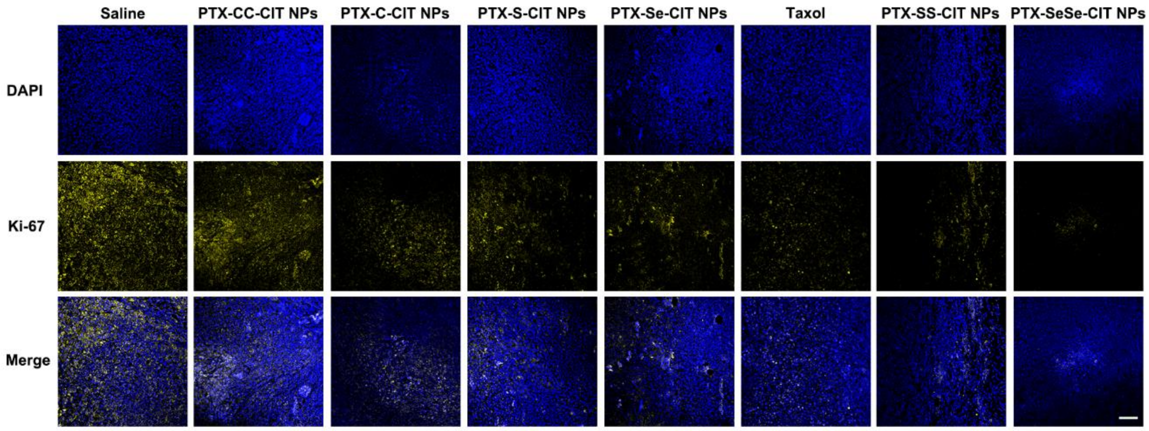

Supplementary Figure 34. Ki-67 immunofluorescence staining. 4T1 tumor sections were prepared after the last treatment. Scale bar represents 50 μm.

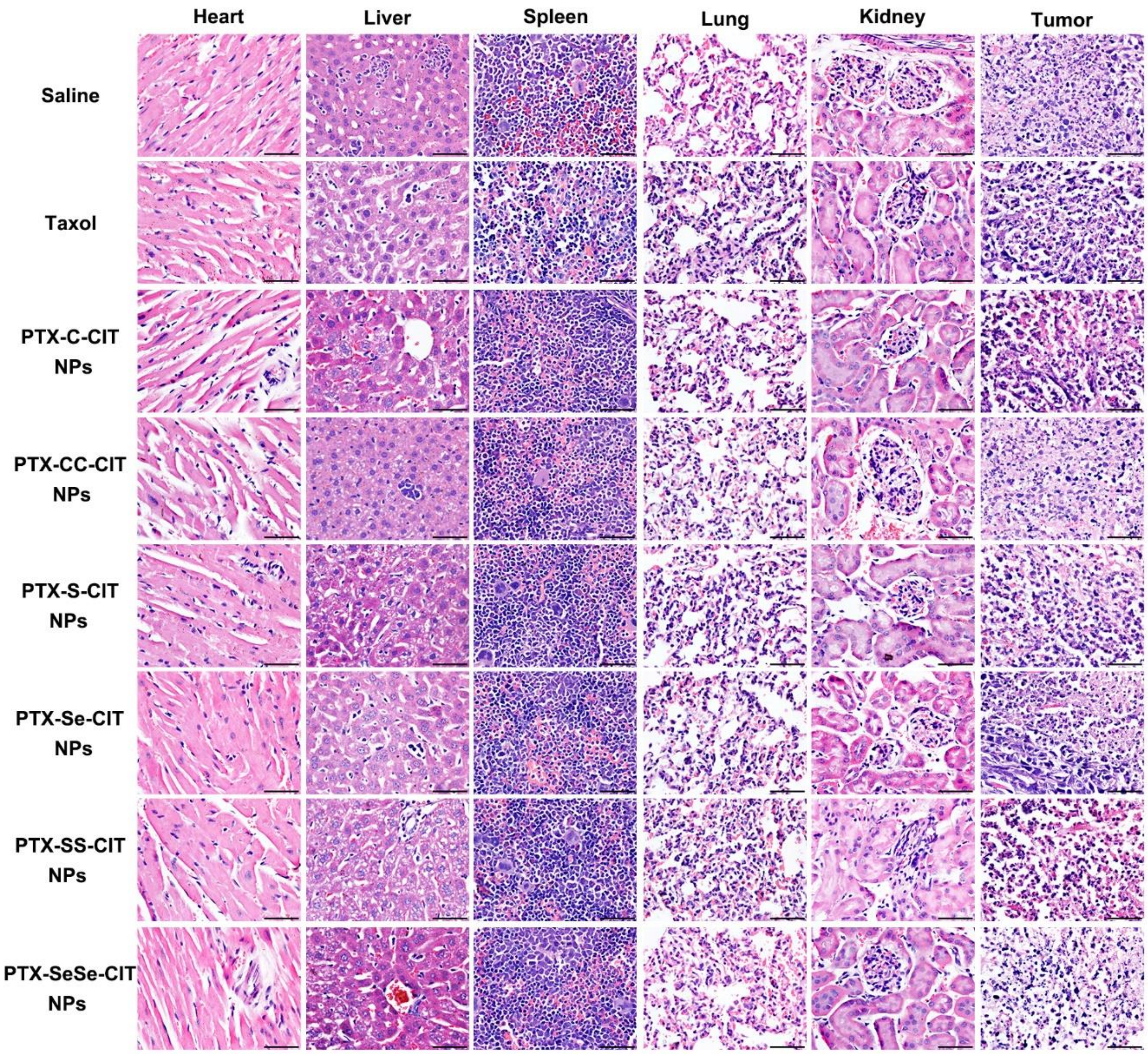

Supplementary Figure 35. H&E staining. The major organs and tumor sections of 4T1 tumor bearing nude mice were prepared after the last treatments. Scale bar represents 50 μm.

Supplementary Tables

Supplementary Table 1. Characterization of PTX-CIT prodrug nanoassemblies without DSPE-PEG_{2K}.

Nanoassemblies	Size (nm)	Size (nm)[a]	Zeta potential (mV)
PTX-S-CIT NPs	95.53±2.25	1191.00±50.69	-16.40±0.32
PTX-SS-CIT NPs	84.30±3.01	776.00±193.70	-18.30±2.49
PTX-Se-CIT NPs	99.09±0.79	803.00±108.00	-18.20±0.76
PTX-SeSe-CIT NPs	82.34±1.63	233.90±7.47	-17.40±1.70
PTX-C-CIT NPs	111.20±1.45	1531.00±12.12	-15.40±0.64
PTX-CC-CIT NPs	107.40±3.00	1631.00±137.40	-19.00±0.97

a: the size of prodrug nanoassemblies after diluted with equal amount of pH 7.4 PBS (containing 10% FBS). Data are presented as mean ± SD (three independent experiments).

Supplementary Table 2. Characterization of PTX-CIT prodrug nanoassemblies with DSPE-PEG_{2K}.

**Nanoassemblies**	**Size (nm)**	**PDI**	**Zeta potential (mV)**	**DL (%)**
PTX-S-CIT NPs	92.07±1.55	0.09±0.07	-21.2±0.40	59.33
PTX-SS-CIT NPs	86.77±1.58	0.05±0.05	-21.5±1.07	57.72
PTX-Se-CIT NPs	88.38±0.67	0.14±0.06	-22.2±4.31	56.96
PTX-SeSe-CIT NPs	86.90±0.04	0.07±0.06	-18.9±3.39	53.39
PTX-C-CIT NPs	99.21±1.83	0.12±0.05	-20.3±1.59	60.27
PTX-CC-CIT NPs	98.96±0.21	0.07±0.01	-19.2±0.40	59.53

Data are presented as mean ± SD (three independent experiments).

Supplementary Table 3. IC_{50} values (nmol L^{-1}) of Taxol and prodrug nanoassemblies against three cell lines (n=3).

Formulations	KB		A549		4T1	
	**48 h**	**72 h**	**48 h**	**72 h**	**48 h**	**72 h**
Taxol	1.8	1.6	22.0	3.6	0.3	1.3
PTX-S-CIT NPs	41.5	26.6	91.4	11.7	209.2	256.8
PTX-SS-CIT NPs	22.2	12.0	59.2	12.1	231.7	180.5
PTX-Se-CIT NPs	14.7	4.7	44.3	2.5	80.9	129.0
PTX-SeSe-CIT NPs	3.3	2.7	53.4	3.8	68.4	89.1
PTX-C-CIT NPs	151.6	116.2	995.0	63.4	744.3	-
PTX-CC-CIT NPs	209.8	123.7	1477.0	113.7	8193.2	-

Supplementary Table 4. Pharmacokinetic parameters of Taxol and prodrug nanoassemblies.

Formulations	Determined [a)]	$AUC_{0\text{-}24h}$ [b)]	$t_{1/2}$ [c)]	MRT [d)]
Taxol	PTX	1.37±0.21	0.86±0.29	0.34±0.06
PTX-S-CIT NPs	PTX-S-CIT	4.04±2.55^{A}	0.13±0.09	0.12±0.16
	PTX	9.76±3.14^{B}	0.12±0.03	0.16±0.13
PTX-SS-CIT NPs	PTX-SS-CIT	8.86±3.19^{B,D,G}	0.16±0.05	0.21±0.08
	PTX	5.19±1.71^{B}	0.33±0.10	0.39±0.03
PTX-Se-CIT NPs	PTS-Se-CIT	4.36±1.36^{B,J}	0.52±0.91	0.21±0.14
	PTX	7.03±2.36^{B,J}	0.19±0.07	0.22±0.07
PTX-SeSe-CIT NPs	PTX-SeSe-CIT	19.20±2.96^{C,F,I}	0.47±0.09	0.55±0.04
	PTX	0.75±0.09^{C,E,H}	1.04±0.41	0.76±0.04
PTX-C-CIT NPs	PTX-C-CIT	8.73±1.39^{C,J}	0.15±0.02	0.18±0.04
	PTX	4.78±0.58^{C,J}	0.22±0.06	0.25±0.01
PTX-CC-CIT NPs	PTX-CC-CIT	9.27±1.60^{C,J}	0.29±0.06	0.35±0.02
	PTX	6.97±1.69^{C,J}	0.29±0.02	0.36±0.03

a) Prodrugs and the released PTX were simultaneously determined. b) Area under the plasma concentration-time curve (nmol h mL^{-1}). c) Half-life (h). d) Mean residence time (h). A: $p < 0.05$, B: $p < 0.01$, C: $p < 0.001$, versus Taxol as the control. D: $p < 0.05$, E: $p < 0.01$, F: $p < 0.001$, versus PTX-Se-CIT NPs as the control. G: $p < 0.05$, H: $p < 0.01$, I: $p < 0.001$, versus PTX-S-CIT NPs as the control. J: $p < 0.001$, versus PTX-SeSe-CIT NPs as the control (Two-tailed Student's t-test). Data are presented as mean ± SD (five independent experiments).

Supplementary Notes

Supplementary Note 1: Characterization of PTX-S-CIT ($C_{63}H_{77}NO_{17}S$)

^{1}H NMR (400 MHz, $CDCl_3$): δ 8.13 (d, 2H, Ar-H, *J*=7.3Hz), 7.75 (d, 2H, Ar-H, *J*=7.4Hz), 7.62 (t, 1H, Ar-H, *J*=7.4Hz), 7.40-7.52 (Ar-H, 10H), 7.02 (d, 1H, *J*=9.2Hz, -NH-), 6.29 (s, 1H, 10-H), 6.25 (t, 1H, *J*=8.6Hz, 13-H), 5.98 (dd, 1H, *J*=3.0Hz, *J*=6.4Hz, 3'-H), 5.69 (d, 1H, *J*=7.0Hz, 2-H), 5.51 (d, *J*=3.5Hz, 1H, H-2', PTX), 5.07 (t, 1H, $(CH_3)_2C\underline{CH}$), 4.99 (d, 1H, *J*=8.0Hz, 5-H), 4.45 (dd, 1H, *J*=6.5Hz, *J*=4.2Hz, 7-H), 4.21 (d, 1H, *J*=8.4Hz, 20α-H), 4.11 (d, 1H, *J*=8.4Hz, 20β-H), 4.09 (m, 2H, $\underline{CH_2}OCO$), 3.82 (d, 1H, *J*=7.0Hz, 3-H), 2.75 (t, 4H, $CH_2\underline{CH_2}S\underline{CH_2}CH_2$), 2.54 (t, 4H, $\underline{CH_2}CH_2SCH_2\underline{CH_2}$), 2.50 (m, 1H, 6α-H), 2.45 (s, 3H, 4-$COCH_3$), 2.34 (m, 2H, 14-H), 2.23 (s, 3H, 10-$COCH_3$), 2.01 (m, 2H, $(CH_3)_2CCH\underline{CH_2}$), 1.95 (t, 1H, 6β-H), 1.94 (s, 3H, 18-CH_3), 1.68 (s, 3H, 19-CH_3), 1.66 (s, 3H, $(\underline{CH_3})_2CCH$, cis-), 1.60 (s, 3H, $(\underline{CH_3})_2CCH$, trans-), 1.59 (m, 2H, $\underline{CH_2}CH_2OCO$), 1.43 (m, 2H, $\underline{CH_2}CH(CH_3)CH_2$), 1.25 (s, 3H, 17-$CH_3$), 1.23 (s, 3H, 16-$CH_3$), 1.23 (m, 1H, $CH_2\underline{CH}(CH_3)CH_2$), 0.90 (d, 3H, $CH_2CH(\underline{CH_3})CH_2$).

^{13}C NMR (400 MHz, DMSO-D6): 202.79(C-9), 171.79(CIT-O$\underline{C}$OCH_2, PTX-O$\underline{C}$OCH_2), 171.32(C'-1), 170.07(C-30), 169.17(C-28), 166.88(C-21), 165.68(C'-5), 139.82(C-12), 134.71(C-38), 133.94(C-11), 133.85(C-25, C-32), 131.93(C-41), 131.04($(CH_3)_2\underline{C}CH$), 130.42(C-22), 130.03(C-23, C-27), 129.15(C-40, C-42), 128.75(C-24, C-26, C-34, C-36), 128.09(C-39, 43), 127.90(C-35, C-33, C-37), 124.95($(CH_3)_2C\underline{C}H$), 84.07(C-5), 80.74(C-4), 77.18(C-1), 75.76(C-20), 75.18(C-10), 74.98(C-2), 74.95(C'-2), 71.30(C-7), 70.87(C-

13), 62.81($\underline{C}H_2OH$), 57.86(C'-3), 55.37($OCOCH_2\underline{C}H_2S\underline{C}H_2CH_2OCO$), 54.37(C-8), 46.55(C-3), 43.42(C-15), 40.14($\underline{C}H_2CH_2OH$), 37.03($\underline{C}H_2CH(CH_3)CH_2$), 36.89(C-6), 35.37(C-14), 34.74-34.47($OCO\underline{C}H_2CH_2SCH_2\underline{C}H_2OCO$), 29.29($CH_2\underline{C}H(CH_3)CH_2$), 26.81(C-17), 25.96($(\underline{C}H_3)_2CCH$, cis-), 25.32($(CH_3)_2CCH\underline{C}H_2$), 23.02(C-16), 21.85(C-29), 21.13(C-31), 19.66($(\underline{C}H_3)_2CCH$, trans-), 17.97($CH_2CH(\underline{C}H_3)CH_2$), 14.35(C-18), 10.24(C-19).

MS (ESI) m/z for $C_{63}H_{77}NO_{17}SNa$ $[M+Na]^+$: 1174.3.

Purity: 99.12%

Supplementary Note 2: Characterization of PTX-SS-CIT ($C_{63}H_{77}NO_{17}S_2$)

^{1}H NMR (400 MHz, $CDCl_3$): δ 8.08 (d, 2H, Ar-H, *J*=7.3Hz), 7.69 (d, 2H, Ar-H, *J*=7.4Hz), 7.55 (t, 1H, Ar-H, *J*=7.4Hz), 7.45-7.33 (Ar-H, 10H), 7.00 (d, 1H, *J*=9.2Hz, -NH-), 6.23 (s, 1H, 10-H), 6.18 (t, 1H, *J*=8.6Hz, 13-H), 5.93 (dd, 1H, *J*=3.0Hz, *J*=6.4Hz, 3'-H), 5.63 (d, 1H, *J*=7.0Hz, 2-H), 5.46 (d, *J*=3.5Hz, 1H, H-2', PTX), 5.00 (t, 1H, $(CH_3)_2C\underline{CH}$), 4.90 (d, 1H, *J*=8.0Hz, 5-H), 4.38 (dd, 1H, *J*=6.5Hz, *J*=4.2Hz, 7-H), 4.24 (d, 1H, *J*=8.4Hz, 20α-H), 4.14 (d, 1H, *J*=8.4Hz, 20β-H), 4.04 (m, 2H, $\underline{CH_2}OCO$), 3.76 (d, 1H, *J*=7.0Hz, 3-H), 2.80 (t, 4H, $CH_2\underline{CH_2}SS\underline{CH_2}CH_2$), 2.60 (t, 4H, $\underline{CH_2}CH_2SSCH_2\underline{CH_2}$), 2.49 (m, 1H, 6α-H), 2.39 (s, 3H, 4-$COCH_3$), 2.29 (m, 2H, 14-H), 2.16 (s, 3H, 10-$COCH_3$), 1.94 (m, 2H, $(CH_3)_2CCH\underline{CH_2}$), 1.87 (t, 1H, 6β-H), 1.87 (s, 3H, 18-CH_3), 1.61 (s, 3H, 19-CH_3), 1.61 (s, 3H, $(\underline{CH_3})_2CCH$, cis-), 1.53 (s, 3H, $(\underline{CH_3})_2CCH$, trans-), 1.52 (m, 2H, $\underline{CH_2}CH_2OCO$), 1.36 (m, 2H, $\underline{CH_2}CH(CH_3)CH_2$), 1.19 (s, 3H, 17-$CH_3$), 1.17 (s, 3H, 16-$CH_3$), 1.12 (m, 1H, $CH_2\underline{CH}(CH_3)CH_2$), 0.84 (d, 3H, $CH_2CH(\underline{CH_3})CH_2$).

^{13}C NMR (400 MHz, DMSO-D6): 202.79(C-9), 171.54(CIT-O$\underline{C}OCH_2$, PTX-O$\underline{C}OCH_2$), 171.05(C'-1), 170.07(C-30), 169.18(C-28), 166.92(C-21), 165.68(C'-5), 139.81(C-12), 134.71(C-38), 133.94(C-11), 133.87(C-25, C-32), 131.93(C-41), 131.05($(CH_3)_2\underline{C}CH$), 130.43(C-22), 130.04(C-23, C-27), 129.16(C-40, C-42), 128.76(C-24, C-26, C-34, C-36), 128.07(C-39, 43), 127.91(C-35, C-33, C-37), 124.95($(CH_3)_2C\underline{C}H$), 84.07(C-5), 80.75(C-4), 77.19(C-1), 75.77(C-20), 75.18(C-10), 75.10(C-2), 74.99(C'-2), 71.34(C-7), 70.87(C-13), 62.94($\underline{C}H_2OH$), 57.86(C'-3), 55.37($OCOCH_2\underline{C}H_2SS\underline{C}H_2CH_2OCO$), 54.36(C-8),

46.55(C-3), 43.42(C-15), 39.48($\underline{C}H_2CH_2OH$), 37.00($\underline{C}H_2CH(CH_3)CH_2$), 36.88(C-6), 35.36(C-14), 33.93-32.72($OCO\underline{C}H_2CH_2SSCH_2\underline{C}H_2OCO$), 29.26($CH_2\underline{C}H(CH_3)CH_2$), 26.82(C-17), 25.96($(\underline{C}H_3)_2CCH$, cis-), 25.32($(CH_3)_2CCH\underline{C}H_2$), 23.03(C-16), 21.85(C-29), 21.12(C-31), 19.65($(\underline{C}H_3)_2CCH$, trans-), 17.98($CH_2CH(\underline{C}H_3)CH_2$), 14.35(C-18), 10.24(C-19).

MS (ESI) m/z for $C_{63}H_{77}NO_{17}S_2Na$ $[M+Na]^+$: 1206.2.

Purity: 99.35%

Supplementary Note 3: Characterization of PTX-Se-CIT ($C_{63}H_{77}NO_{17}Se$)

^{1}H NMR (400 MHz, $CDCl_3$): δ 8.08 (d, 2H, Ar-H, *J*=7.3Hz), 7.70 (d, 2H, Ar-H, *J*=7.4Hz), 7.55 (t, 1H, Ar-H, *J*=7.4Hz), 7.46-7.34 (Ar-H, 10H), 6.97 (d, 1H, *J*=9.2Hz, -NH-), 6.22 (s, 1H, 10-H), 6.18 (t, 1H, *J*=8.6Hz, 13-H), 5.90 (dd, 1H, *J*=3.0Hz, *J*=6.4Hz, 3'-H), 5.62 (d, 1H, *J*=7.0Hz, 2-H), 5.45 (d, *J*=3.5Hz, 1H, H-2', PTX), 5.00 (t, 1H, $(CH_3)_2C\underline{CH}$), 4.91 (d, 1H, *J*=8.0Hz, 5-H), 4.39 (dd, 1H, *J*=6.5Hz, *J*=4.2Hz, 7-H), 4.24 (d, 1H, *J*=8.4Hz, 20α-H), 4.14 (d, 1H, *J*=8.4Hz, 20β-H), 4.04 (m, 2H, $\underline{CH_2}OCO$), 3.75 (d, 1H, *J*=7.0Hz, 3-H), 2.69 (t, 4H, $CH_2\underline{CH_2}Se\underline{CH_2}CH_2$), 2.59 (t, 4H, $\underline{CH_2}CH_2SeCH_2\underline{CH_2}$), 2.49 (m, 1H, 6α-H), 2.39 (s, 3H, 4-$COCH_3$), 2.28 (m, 2H, 14-H), 2.16 (s, 3H, 10-$COCH_3$), 1.91 (m, 2H, $(CH_3)_2CCH\underline{CH_2}$), 1.89 (t, 1H, 6β-H), 1.86 (s, 3H, 18-CH_3), 1.68 (s, 3H, 19-CH_3), 1.61 (s, 3H, $(\underline{CH_3})_2CCH$, cis-), 1.53 (s, 3H, $(\underline{CH_3})_2CCH$, trans-), 1.52 (m, 2H, $\underline{CH_2}CH_2OCO$), 1.36 (m, 2H, $\underline{CH_2}CH(CH_3)CH_2$), 1.19 (s, 3H, 17-$CH_3$), 1.16 (s, 3H, 16-$CH_3$), 1.07 (m, 1H, $CH_2\underline{CH}(CH_3)CH_2$), 0.850 (d, 3H, $CH_2CH(\underline{CH_3})CH_2$).

^{13}C NMR (400 MHz, DMSO-D6): 202.80(C-9), 172.14(CIT-O$\underline{C}OCH_2$, PTX-O$\underline{C}OCH_2$), 171.66(C'-1), 170.07(C-30), 169.17(C-28), 166.88(C-21), 165.68(C'-5), 139.83(C-12), 134.71(C-38), 133.94(C-11), 133.84(C-25, C-32), 131.93(C-41), 131.04($(CH_3)_2\underline{C}CH$), 130.42(C-22), 130.03(C-23, C-27), 129.15(C-40, C-42), 128.76(C-24, C-26, C-34, C-36), 128.09(C-39, 43), 127.90(C-35, C-33, C-37), 124.96($(CH_3)_2C\underline{C}H$), 84.08(C-5), 80.74(C-4), 77.18(C-1), 75.76(C-20), 75.18(C-10), 75.10(C-2), 74.98(C'-2), 71.30(C-7), 70.87(C-13), 62.81($\underline{C}H_2OH$), 57.86(C'-3), 55.37($OCOCH_2\underline{C}H_2Se\underline{C}H_2CH_2OCO$), 54.39(C-8),

46.55(C-3), 43.42(C-15), 40.14($\underline{C}H_2CH_2OH$), 37.03($\underline{C}H_2CH(CH_3)CH_2$), 36.89(C-6), 35.66(C-14), 35.38-34.89($OCO\underline{C}H_2CH_2SeCH_2\underline{C}H_2OCO$), 29.28($CH_2\underline{C}H(CH_3)CH_2$), 26.81(C-17), 25.96($(\underline{C}H_3)_2CCH$, cis-), 25.32($(CH_3)_2CCH\underline{C}H_2$), 23.02(C-16), 21.85(C-29), 21.13(C-31), 19.66($(\underline{C}H_3)_2CCH$, trans-), 17.98($CH_2CH(\underline{C}H_3)CH_2$), 14.37(C-18), 10.24(C-19).

MS (ESI) m/z for $C_{63}H_{77}NO_{17}SeNa$ $[M+Na]^+$: 1222.4.

Purity: 99.03%

Supplementary Note 4: Characterization of PTX-SeSe-CIT ($C_{63}H_{77}NO_{17}Se_2$)

^1H NMR (400 MHz, $CDCl_3$): δ 8.09 (d, 2H, Ar-H, *J*=7.3Hz), 7.69 (d, 2H, Ar-H, *J*=7.4Hz), 7.55 (t, 1H, Ar-H, *J*=7.4Hz), 7.45-7.33 (Ar-H, 10H), 7.00 (d, 1H, *J*=9.2Hz, -NH-), 6.23 (s, 1H, 10-H), 6.19 (t, 1H, *J*=8.6Hz, 13-H), 5.91 (dd, 1H, *J*=3.0Hz, *J*=6.4Hz, 3'-H), 5.62 (d, 1H, *J*=7.0Hz, 2-H), 5.46 (d, *J*=3.5Hz, 1H, H-2', PTX), 5.01 (t, 1H, $(CH_3)_2C\underline{CH}$), 4.90 (d, 1H, *J*=8.0Hz, 5-H), 4.39 (dd, 1H, *J*=6.5Hz, *J*=4.2Hz, 7-H), 4.24 (d, 1H, *J*=8.4Hz, 20α-H), 4.14 (d, 1H, *J*=8.4Hz, 20β-H), 4.02 (m, 2H, $\underline{CH_2}OCO$), 3.76 (d, 1H, *J*=7.0Hz, 3-H), 2.98 (t, 4H, $CH_2\underline{CH_2}SeSe\underline{CH_2}CH_2$), 2.68 (t, 4H, $\underline{CH_2}CH_2SeSeCH_2\underline{CH_2}$), 2.50 (m, 1H, 6α-H), 2.39 (s, 3H, 4-$COCH_3$), 2.29 (m, 2H, 14-H), 2.16 (s, 3H, 10-$COCH_3$), 1.93 (m, 2H, $(CH_3)_2CCH\underline{CH_2}$), 1.88 (t, 1H, 6β-H), 1.86 (s, 3H, 18-CH_3), 1.68 (s, 3H, 19-CH_3), 1.61 (s, 3H, $(\underline{CH_3})_2CCH$, cis-), 1.53 (s, 3H, $(\underline{CH_3})_2CCH$, trans-), 1.52 (m, 2H, $\underline{CH_2}CH_2OCO$), 1.36 (m, 2H, $\underline{CH_2}CH(CH_3)CH_2$), 1.19 (s, 3H, 17-$CH_3$), 1.17 (s, 3H, 16-$CH_3$), 1.07 (m, 1H, $CH_2\underline{CH}(CH_3)CH_2$), 0.84 (d, 3H, $CH_2CH(\underline{CH_3})CH_2$).

^{13}C NMR (400 MHz, DMSO-D6): 202.80(C-9), 171.90(CIT-O$\underline{C}OCH_2$, PTX-O$\underline{C}OCH_2$), 171.38(C'-1), 170.08(C-30), 169.17(C-28), 166.91(C-21), 165.68(C'-5), 139.84(C-12), 134.69(C-38), 133.93(C-11), 133.85(C-25, C-32), 131.92(C-41), 131.04($(CH_3)_2\underline{C}CH$), 130.43(C-22), 130.04(C-23, C-27), 129.15(C-40, C-42), 128.74(C-24, C-26, C-34, C-36), 128.11(C-39, 43), 127.94(C-35, C-33, C-37), 124.94($(CH_3)_2C\underline{C}H$), 84.08(C-5), 80.73(C-4), 77.19(C-1), 75.76(C-20), 75.29(C-10), 75.21(C-2), 74.98(C'-2), 71.34(C-7), 70.87(C-13), 62.92($\underline{C}H_2OH$), 57.86(C'-3), 55.29($OCOCH_2\underline{C}H_2SeSe\underline{C}H_2CH_2OCO$), 54.42(C-8),

46.55(C-3), 43.43(C-15), 39.48($\underline{C}H_2CH_2OH$), 37.00($\underline{C}H_2CH(CH_3)CH_2$), 36.89(C-6), 35.39(C-14), 34.88-34.57($OCO\underline{C}H_2CH_2SSCH_2\underline{C}H_2OCO$), 29.27($CH_2\underline{C}H(CH_3)CH_2$), 26.82(C-17), 25.96($(\underline{C}H_3)_2CCH$, cis-), 25.33($(CH_3)_2CCH\underline{C}H_2$), 23.06(C-16), 21.85(C-29), 21.12(C-31), 19.64($(\underline{C}H_3)_2CCH$, trans-), 17.97($CH_2CH(\underline{C}H_3)CH_2$), 14.38(C-18), 10.24(C-19).

MS (ESI) m/z for $C_{63}H_{77}NO_{17}Se_2Na$ $[M+Na]^+$: 1300.8.

Purity: 99.41%

Supplementary Note 5: Characterization of PTX-C-CIT ($C_{64}H_{79}NO_{17}$)

^{1}H NMR (400 MHz, $CDCl_3$): δ 8.08 (d, 2H, Ar-H, *J*=7.3Hz), 7.66 (d, 2H, Ar-H, *J*=7.4Hz), 7.54 (t, 1H, Ar-H, *J*=7.4Hz), 7.45-7.34 (Ar-H, 10H), 6.84 (d, 1H, *J*=9.2Hz, -NH-), 6.23 (s, 1H, 10-H), 6.19 (t, 1H, *J*=8.6Hz, 13-H), 5.90 (dd, 1H, *J*=3.0Hz, *J*=6.4Hz, 3'-H), 5.62 (d, 1H, *J*=7.0Hz, 2-H), 5.43 (d, *J*=3.5 Hz, 1H, H-2’, PTX), 5.01 (t, 1H, $(CH_3)_2C\underline{CH}$), 4.91 (d, 1H, *J*=8.0Hz, 5-H), 4.38 (dd, 1H, *J*=6.5Hz, *J*=4.2Hz, 7-H), 4.24 (d, 1H, *J*=8.4Hz, 20α-H), 4.14 (d, 1H, *J*=8.4Hz, 20β-H), 4.02 (m, 2H, $\underline{CH_2}OCO$), 3.75 (d, 1H, *J*=7.0Hz, 3-H), 2.49 (m, 1H, 6α-H), 2.39 (s, 3H, 4-$COCH_3$), 2.26 (m, 2H, 14-H), 2.20 (t, 4H, $\underline{CH_2}CH_2CH_2CH_2\underline{CH_2}$), 2.16 (s, 3H, 10-$COCH_3$), 1.91 (m, 2H, $(CH_3)_2CCH\underline{CH_2}$), 1.89 (t, 1H, 6β-H), 1.87 (s, 3H, 18-CH_3), 1.68 (s, 3H, 19-CH_3), 1.61 (s, 3H, $(\underline{CH_3})_2CCH$, cis-), 1.53 (s, 3H, $(\underline{CH_3})_2CCH$, trans-), 1.52 (m, 2H, $\underline{CH_2}CH_2OCO$), 1.50 (t, 4H, $CH_2\underline{CH_2}$ $CH_2\underline{CH_2}CH_2$), 1.36 (m, 2H, $\underline{CH_2}CH(CH_3)CH_2$), 1.20 (s, 3H, 17-$CH_3$), 1.19 (t, 2H, $CH_2CH_2\underline{CH_2}CH_2CH_2$), 1.16 (s, 3H, 16-$CH_3$), 1.12 (m, 1H, $CH_2\underline{CH}(CH_3)CH_2$), 0.85 (d, 3H, $CH_2CH(\underline{CH_3})CH_2$).

^{13}C NMR (400 MHz, DMSO-D6): 202.78(C-9), 173.15(CIT-O$\underline{C}$OCH_2, PTX-O$\underline{C}$OCH_2), 172.74(C'-1), 170.09(C-30), 169.17(C-28), 166.79(C-21), 165.67(C'-5), 139.81(C-12), 134.68(C-38), 133.93(C-11), 133.87(C-25, C-32), 131.93(C-41), 131.05($(CH_3)_2\underline{C}CH$), 130.44(C-22), 130.03(C-23, C-27), 129.14(C-40, C-42), 128.76(C-24, C-26, C-34, C-36), 128.09(C-39, 43), 127.85(C-35, C-33, C-37), 124.94($(CH_3)_2C\underline{C}H$), 84.08(C-5), 80.74(C-4), 77.19(C-1), 75.76(C-20), 75.18(C-10), 74.98(C-2), 74.95(C'-2), 71.29(C-7), 70.86(C-

13), 62.44($\underline{C}H_2OH$), 57.86(C'-3), 54.42(C-8), 46.54(C-3), 43.42(C-15), 40.14($\underline{C}H_2CH_2OH$), 37.03($\underline{C}H_2CH(CH_3)CH_2$), 36.89(C-6), 35.42(C-14), 33.87-33.47($OCO\underline{C}H_2CH_2CH_2CH_2\underline{C}H_2OCO$), 29.32($CH_2\underline{C}H(CH_3)CH_2$), 28.35-28.18($OCOCH_2\underline{C}H_2\underline{C}H_2\underline{C}H_2CH_2OCO$), 26.81(C-17), 25.95($(\underline{C}H_3)_2CCH$, cis-), 25.32($(CH_3)_2CCH\underline{C}H_2$), 23.00(C-16), 21.86(C-29), 21.12(C-31), 19.67($(\underline{C}H_3)_2CCH$, trans-), 17.96($CH_2CH(\underline{C}H_3)CH_2$), 14.30(C-18), 10.24(C-19).

MS (ESI) m/z for $C_{64}H_{79}NO_{17}Na$ $[M+Na]^+$: 1156.6.

Purity: 98.84%

Supplementary Note 6: Characterization of PTX-CC-CIT ($C_{65}H_{81}NO_{17}$)

^{1}H NMR (400 MHz, $CDCl_3$): δ 8.08 (d, 2H, Ar-H, *J*=7.3Hz), 7.68 (d, 2H, Ar-H, *J*=7.4Hz), 7.54 (t, 1H, Ar-H, *J*=7.4Hz), 7.45-7.34 (Ar-H, 10H), 6.85 (d, 1H, *J*=9.2Hz, -NH-), 6.23 (s, 1H, 10-H), 6.19 (t, 1H, *J*=8.6Hz, 13-H), 5.90 (dd, 1H, *J*=3.0Hz, *J*=6.4Hz, 3'-H), 5.62 (d, 1H, *J*=7.0Hz, 2-H), 5.44 (d, *J*=3.5Hz, 1H, H-2’, PTX), 5.01 (t, 1H, $(CH_3)_2C\underline{CH}$), 4.92 (d, 1H, *J*=8.0Hz, 5-H), 4.39 (dd, 1H, *J*=6.5Hz, *J*=4.2Hz, 7-H), 4.24 (d, 1H, *J*=8.4Hz, 20α-H), 4.14 (d, 1H, *J*=8.4Hz, 20β-H), 4.01 (m, 2H, $\underline{CH_2}OCO$), 3.75 (d, 1H, *J*=7.0Hz, 3-H), 2.50 (m, 1H, 6α-H), 2.39 (s, 3H, 4-$COCH_3$), 2.31 (m, 2H, 14-H), 2.20 (t, 4H, $\underline{CH_2}CH_2CH_2CH_2CH_2\underline{CH_2}$), 2.18(s, 3H, 10-$COCH_3$), 2.09 (m, 2H, $(CH_3)_2CCH\underline{CH_2}$), 1.87 (t, 1H, 6β-H), 1.82 (s, 3H, 18-CH_3), 1.68 (s, 3H, 19-CH_3), 1.61 (s, 3H, $(\underline{CH_3})_2CCH$, cis-), 1.53 (s, 3H, $(\underline{CH_3})_2CCH$, trans-), 1.52 (m, 2H, $\underline{CH_2}CH_2OCO$), 1.50 (t, 4H, $CH_2\underline{CH_2}$ $CH_2CH_2\underline{CH_2}CH_2$), 1.36 (m, 2H, $\underline{CH_2}CH(CH_3)CH_2$), 1.20 (s, 3H, 17-$CH_3$), 1.19 (t, 4H, $CH_2CH_2\underline{CH_2}\underline{CH_2}CH_2CH_2$), 1.16 (s, 3H, 16-$CH_3$), 1.12 (m, 1H, $CH_2\underline{CH}(CH_3)CH_2$), 0.85 (d, 3H, $CH_2CH(\underline{CH_3})CH_2$).

^{13}C NMR (400 MHz, DMSO-D6): 202.77(C-9), 173.25(CIT-O$\underline{C}$OCH_2, PTX-O$\underline{C}$OCH_2), 172.82(C'-1), 170.10(C-30), 169.17(C-28), 166.76(C-21), 165.67(C'-5), 139.79(C-12), 134.67(C-38), 133.88(C-11), 133.88(C-25, C-32), 131.93(C-41), 131.05($(CH_3)_2\underline{C}CH$), 130.44(C-22), 130.03(C-23, C-27), 129.14(C-40, C-42), 128.75(C-24, C-26, C-34, C-36), 128.10(C-39, 43), 127.85(C-35, C-33, C-37), 124.94($(CH_3)_2C\underline{C}H$), 84.08(C-5), 80.74(C-4), 77.19(C-1), 75.76(C-20), 75.17(C-10), 74.99(C-2), 74.93(C'-2), 71.29(C-7), 70.86(C-

13), 62.41($\underline{C}H_2OH$), 57.86(C'-3), 54.44(C-8), 46.55(C-3), 43.42(C-15), 40.14($\underline{C}H_2CH_2OH$), 37.03($\underline{C}H_2CH(CH_3)CH_2$), 36.88(C-6), 35.43(C-14), 33.94-33.56($OCO\underline{C}H_2CH_2CH_2CH_2CH_2\underline{C}H_2OCO$), 29.30($CH_2\underline{C}H(CH_3)CH_2$), 28.49-28.38($OCOCH_2\underline{C}H_2\underline{C}H_2\underline{C}H_2\underline{C}H_2CH_2OCO$), 26.81(C-17), 25.95($(\underline{C}H_3)_2CCH$, cis-), 25.32($(CH_3)_2CCH\underline{C}H_2$), 23.01(C-16), 21.86(C-29), 21.12(C-31), 19.67($(\underline{C}H_3)_2CCH$, trans-), 17.96($CH_2CH(\underline{C}H_3)CH_2$), 14.30(C-18), 10.24(C-19).

MS (ESI) m/z for $C_{65}H_{81}NO_{17}Na$ $[M+Na]^+$: 1170.8.

Purity: 99.38%
