# Supplementary material for: Probing the impact of sulfur/selenium/carbon linkages on prodrug nanoassemblies for cancer therapy
Source: Nat Commun. 2019 Jul 19;10:3211. doi: 10.1038/s41467-019-11193-x (PMC6642185; doi:10.1038/s41467-019-11193-x)
Supplement: Supplementary file 2 — Reporting Summary [file 41467_2019_11193_MOESM2_ESM.pdf]

nature research

Corresponding author(s): Jin Sun

Last updated by author(s): Jun 13, 2019

Reporting Summary

Nature Research wishes to improve the reproducibility of the work that we publish. This form provides structure for consistency and transparency in reporting. For further information on Nature Research policies, see Authors & Referees and the Editorial Policy Checklist.

Statistics

For all statistical analyses, confirm that the following items are present in the figure legend, table legend, main text, or Methods section.

n/a	Confirmed	
☐	☒	The exact sample size (n) for each experimental group/condition, given as a discrete number and unit of measurement
☐	☒	A statement on whether measurements were taken from distinct samples or whether the same sample was measured repeatedly
☐	☒	The statistical test(s) used AND whether they are one- or two-sided<br>*Only common tests should be described solely by name; describe more complex techniques in the Methods section.*
☐	☒	A description of all covariates tested
☐	☒	A description of any assumptions or corrections, such as tests of normality and adjustment for multiple comparisons
☐	☒	A full description of the statistical parameters including central tendency (e.g. means) or other basic estimates (e.g. regression coefficient) AND variation (e.g. standard deviation) or associated estimates of uncertainty (e.g. confidence intervals)
☐	☒	For null hypothesis testing, the test statistic (e.g. F, t, r) with confidence intervals, effect sizes, degrees of freedom and P value noted<br>*Give P values as exact values whenever suitable.*
☐	☒	For Bayesian analysis, information on the choice of priors and Markov chain Monte Carlo settings
☐	☒	For hierarchical and complex designs, identification of the appropriate level for tests and full reporting of outcomes
☐	☒	Estimates of effect sizes (e.g. Cohen's d, Pearson's r), indicating how they were calculated

Our web collection on statistics for biologists contains articles on many of the points above.

Software and code

Policy information about availability of computer code

Data collection	ChemDraw Ultra 12.0; Materials Studio 2017, Sybyl 6.9.1, AutoDock 4.0; HPLC: Empower 3; UPLC-MS: MassLynx V4.1; Malvern: Zetasizer Software 7.01; Microplate reader: SkanIt 2.4.3.37; Confocal laser scanning microscopy: NIS 4.13; Flow Cytometer: FACSComp TM 6.1
Data analysis	MestReC 4.9.9.9; OriginPro 9.0, IBM SPSS Statistics 20; Flowjo_V10; DAS 2.1.1; Microsoft Office 2017;

For manuscripts utilizing custom algorithms or software that are central to the research but not yet described in published literature, software must be made available to editors/reviewers. We strongly encourage code deposition in a community repository (e.g. GitHub). See the Nature Research guidelines for submitting code & software for further information.

Data

Policy information about availability of data

All manuscripts must include a data availability statement. This statement should provide the following information, where applicable:

- Accession codes, unique identifiers, or web links for publicly available datasets
- A list of figures that have associated raw data
- A description of any restrictions on data availability

All relevant data are available from the authors.

Field-specific reporting

Please select the one below that is the best fit for your research. If you are not sure, read the appropriate sections before making your selection.

☒ Life sciences ☐ Behavioural & social sciences ☐ Ecological, evolutionary & environmental sciences

Life sciences study design

All studies must disclose on these points even when the disclosure is negative.

Sample size	For in vitro study, sample size was three to satisfy statistical analysis. For in vivo study, sample size was five to eliminate the effect of individual difference.
Data exclusions	No data were excluded from the analyses.
Replication	The methods in this study were mature and reliable, and have been widely used and verified in our previous study. These could insure the reproducibility of the results.
Randomization	Samples were randomly allocated into experimental groups.
Blinding	The investigators were blinded to group allocation during data collection and analysis.

Reporting for specific materials, systems and methods

We require information from authors about some types of materials, experimental systems and methods used in many studies. Here, indicate whether each material, system or method listed is relevant to your study. If you are not sure if a list item applies to your research, read the appropriate section before selecting a response.

Materials & experimental systems

n/a	Involved in the study	
☐	☒	Antibodies
☐	☒	Eukaryotic cell lines
☒	☐	Palaeontology
☐	☒	Animals and other organisms
☒	☐	Human research participants
☒	☐	Clinical data

Methods

n/a	Involved in the study	
☒	☐	ChIP-seq
☐	☒	Flow cytometry
☒	☐	MRI-based neuroimaging

Antibodies

Antibodies used	Ki67 polyclonal antibody (ABclonal, A2094); goat anti-rabbit antibody (BOSTER, SA1064)
Validation	The species and application of primary antibody is validated by ABclonal.

Eukaryotic cell lines

Policy information about cell lines

Cell line source(s)	KB cells, A549 cells and 4T1 cells were obtained from COBIOER Biotechnology Co., Ltd (Nanjing, China).
Authentication	KB cells and A549 cells were authenticated using STR profiling. Mouse breast carcinoma 4T1 cells were not authenticated.
Mycoplasma contamination	All cell lines tested negative for mycoplasma contamination.
Commonly misidentified lines (See ICLAC register)	KB cells are suspected contaminated by Hela cells, and are listed in the database of commonly misidentified cell lines by International Cell Line Authentication Committee (ICLAC). In this study, KB cells were just used to evaluated the in vitro cytotoxicity and in vivo antitumor efficiency of prodrug nanoassemblies. Hela cells are also widely used to evaluated the cytotoxicity and antitumor efficiency of chemotherapeutics drugs, and Hela cells are also very sensitive to PTX. Therefore, being used as an evaluation model instead of a disease model, the potential contamination of KB cells by Hela cells might not significantly influence the pharmacodynamic evaluation of prodrug nanoassemblies under the same experimental conditions. Moreover, the in vitro cytotoxicity of prodrug nanoassemblies were further validated using A549 cells and 4T1 cells, and the in vivo antitumor efficiency of prodrug nanoassemblies were further investigated using a 4T1 tumor bearing BALB/c mice.

Animals and other organisms

Policy information about studies involving animals; ARRIVE guidelines recommended for reporting animal research

Laboratory animals	Male Sprague-Dawley rats; Female BALB/c mice; Female nude mice.

Wild animals	The study did not involve wild animals.
Field-collected samples	The study did not involve samples collected from the field.
Ethics oversight	All the animal experiments were conducted according to the Guidelines for the Care and Use of Laboratory Animals approved by the Institutional Animal Ethical Care Committee (IAEC) of Shenyang Pharmaceutical University.

Note that full information on the approval of the study protocol must also be provided in the manuscript.

Flow Cytometry

Plots

Confirm that:

☒ The axis labels state the marker and fluorochrome used (e.g. CD4-FITC).

☒ The axis scales are clearly visible. Include numbers along axes only for bottom left plot of group (a 'group' is an analysis of identical markers).

☒ All plots are contour plots with outliers or pseudocolor plots.

☒ A numerical value for number of cells or percentage (with statistics) is provided.

Methodology

Sample preparation	Cells were obtained from COBIOER Biotechnology Co., Ltd (Nanjing, China).
Instrument	BD FACSCalibur (E20200235)
Software	FACSComp TM 6.1; Flowjo_V10
Cell population abundance	Only KB cells was used for analysis.
Gating strategy	Rectangle gates based on FSC-H and SSC-H signals was used to exclude cell debris and clumps.

☒ Tick this box to confirm that a figure exemplifying the gating strategy is provided in the Supplementary Information.
